# Ethnomedicinal, phytochemical and pharmacological investigations of *Baccharis dracunculifolia* DC. (ASTERACEAE)

**DOI:** 10.3389/fphar.2022.1048688

**Published:** 2022-11-28

**Authors:** Zilda Cristiani Gazim, Juliana Silveira Valle, Isabela Carvalho dos Santos, Isabelle Luiz Rahal, Gabriela Catuzo Canonico Silva, Ana Daniela Lopes, Suelen Pereira Ruiz, Maria Graciela Iecher Faria, Ranulfo Piau Junior, Daniela Dib Gonçalves

**Affiliations:** ^1^ Chemistry Laboratory of Natural Products, Graduate Program in Animal Science and Biotechnology Applied to Agriculture, Paranaense University, Umuarama, Brazil; ^2^ Preventive Veterinary Medicine and Public Health Laboratory, Postgraduate Program in Animal Science with an Emphasis on Bioactive Products, Paranaense University, Umuarama, Brazil; ^3^ Molecular Biology Laboratory, Graduate Program in Animal Science and Biotechnology Applied to Agriculture, Paranaense University, Umuarama, Brazil; ^4^ Agricultural Microbiology and Nematology Laboratory, Graduate Program in Biotechnology Applied to Agriculture, Paranaense University, Umuarama, Brazil; ^5^ Laboratory of Biotechnology of Plant Products and Microorganisms, Postgraduate Program in Biotechnology Applied to Agriculture, Paranaense University, Umuarama, Brazil

**Keywords:** artepillin C, baccharin, ethnopharmacology, green propolis, nerolidol, sphatulenol, traditional folk medicine

## Abstract

*Baccharis dracunculifolia* DC (Lamiaceae) (Asteraceae) is found in South America, mainly in Argentina, Brazil, Bolivia, Paraguay and Uruguay. Folk medicine is used as a sedative, hypotensive, bronchodilator, cardiovascular disorders, anti-flu, and also in skin wounds. Considered the main source of green propolis, which increases the pharmacological interest in this species. It is also known as a “benefactor” plant facilitating the development of other plant species around it, being indicated for the recovery of degraded areas. This species has been studied for decades in order to isolate and identify the active principles present in the aerial parts (leaves and flowers) and roots. The present study consists of a review of the scientific literature addressing the ethnobotanical, ethnomedicinal, phytochemical, pharmacological and potential cytotoxic effects of the *B. dracunculifolia* species. In this survey, we sought to investigate issues related to the botanical and geographic description of the species, the ethnobotanical uses, as well as the phytochemical studies of the essential oil, extracts and green propolis obtained from the aerial parts and roots of *B. dracunculifolia*. Using high precision analytical tools, numerous compounds have already been isolated and identified from leaves and flowers such as the flavonoids: naringenin, acacetin, dihydrokaempferol, isosakuranetin and kaempferide; phenolic acids: *p*-coumaric, dihydrocoumaric, ferulic (E)-cinnamic, hydroxycinnamic, gallic, caffeic, and several caffeoylquinic acids derivatives; phenolic acids prenylated: artepillin C, baccharin, drupanin; the glycosides dracuculifosides and the pentacyclic triterpenoids: *Baccharis* oxide and friedelanol. The predominant class in the essential oil of leaves and flowers are terpenoids comprising oxygenated monoterpenes and sesquiterpenes, highlighting the compounds nerolidol, spathulenol, germacrene D and bicyclogermacrene. These compounds give the species high antimicrobial, antioxidant, antitumor, analgesic, immunomodulatory and antiparasitic potential, making this species a promising herbal medicine. *In vitro* toxicity assays with *B. dracunculifolia* extract showed low or no cytotoxicity. However, *in vivo* analyses with high doses of the aqueous extract resulted in genotoxic effects, which leads us to conclude that the toxicity of this plant is dose-dependent.

## Graphical Abstract

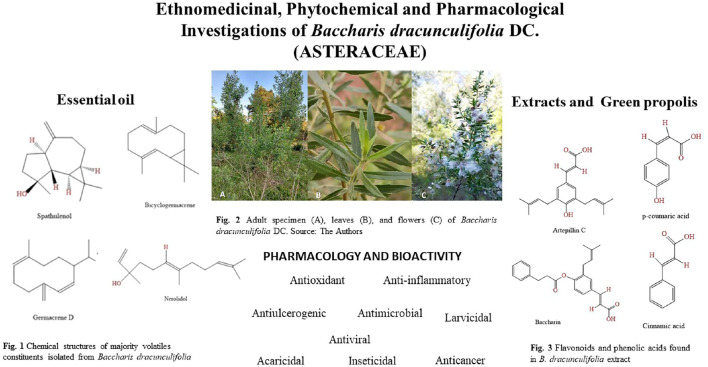

## 1 Introduction

*B. dracunculifolia* is a medicinal bush widely distributed in South America. In Brazil, it is popularly known as “Vassourinha” or “Alecrim do Campo”, it is a source of a natural resinous substance produced by bees (*Apis mellifera*), called green propolis (Belini et al., 2016). Due to its rapid vegetative development, this species is widely used in the restoration of degraded areas, acting as a “nurse species” facilitating the development of other plant species (“beneficiaries”). This benefit occurred in areas where *B. dracunculifolia* were planted, indicating improvements in conditions of temperature, humidity and availability of organic matter in the soil (Siqueira et al., 2022). In folk medicine, tea made from the leaves is used for liver and stomach problems (Bonin et al., 2020). In addition, the infusion and decoction of the flowers are widely used in alternative medicine to treat inflammatory processes as well as liver disorders and stomach ulcers (Salazar et al., 2018).

Research carried out with *B. dracunculifolia* indicates biological activities such as anti-inflammatory (Santos et al., 2010; Brandenburg et al., 2020; França et al., 2022), antiulcerogenic (Klopell et al., 2007; Lemos et al., 2007; Massignani et al., 2009; Costa et al., 2019; Boeing et al., 2021), immunomodulatory (Burgos et al., 2022), neuroprotective (França et al., 2022), antioxidant (Veiga et al., 2017; Casagrande et al., 2018; Tomazzoli et al., 2021; Luchesi et al., 2022; Monteiro et al., 2022); antimicrobial (Veiga et al., 2017; Casagrande et al., 2018; Salazar et al., 2018; Cazella et al., 2019; Pedrotti et al., 2019; Bonin et al., 2020; Timbé et al., 2021; Barbosa et al., 2022; Bernardes et al., 2022; Monteiro et al., 2022), anti-phytopathogenic (Luchesi et al., 2022), insecticide and acaricide (Chaaban et al., 2018; Cazella et al., 2020).

The biological potential found in this species is due to the presence of phenolic and terpenoid compounds. The most common phenolic acids occur in prenylated form, highlighting drupanin, artepillin C, baccharin and baccharin-5″-aldehyde (Minteguiaga et al., 2022). The flavonoids found in aerial parts of *B. dracunculifolia* are naringenin, acacetin, dihydrokaempferol, isosakuranetin and kaempferide (Nagatani et al., 2002a), and are often reported as major constituents of green propolis (Bonin et al., 2020).

The essential oil present in the leaves and flowers is colorless with a characteristic odor of honey (Cazella et al., 2019), being highly valued in the perfumery industry. Trans-nerolidol as the major compound of the essential oil, is regulated by the Food and Drug Administration (FDA) and is used as a flavoring agent in the food industry (Belini et al., 2016). As for the chemical composition, it consists of terpenoids, with the majority class of oxygenated sesquiterpenes and hydrocarbons (Cazella et al., 2019; Minteguiaga et al., 2022).

Thus, this review aims to address the ethnobotanical, ethnomedicinal, phytochemical and pharmacological aspects of the essential oil, extracts and green propolis of *B. dracunculifolia.*

## 2 Asteraceae family

The Asteraceae family is one of the largest flowering plant families, including over 1,600 genera and 25,000 species worldwide. This family includes known species of medicinal importance such as wormwood, chamomile and dandelion. Most species that make up the Asteraceae family have a long history in traditional medicine; some members have been cultivated for over 3,000 years for medicinal and food purposes. They have a wide range of pharmacological activities, with emphasis on anti-inflammatory, antimicrobial, antioxidant and hepatoprotective activities (Rolnik and Olas, 2021).

The Asteraceae are distributed throughout the world, with the exception of Antarctica, with habitats ranging from forests to savanas. Regarding morphology, some species are trees reaching more than 30 m, others are shrubs such as *B. dracunculifolia* and most species are perennial herbs (Bohm and Stuessy, 2001).

### 2.1 Botanical description, geographic distribution

*B. dracunculifolia* is a medicinal ethnobotanical plant belonging to the flora of the Americas, mainly South America. In this continent it is known as an aromatic plant. From the aerial parts, the essential oil is extracted and used in the perfumery industry, in addition to its known therapeutic potential (Minteguiaga et al., 2022). Furthermore, this species provides many services to the ecosystem, including assisting in the formation of a rich fauna of insects and different pollinating species essential for the functioning and sustainability of ecosystems (Fernandes et al., 2018).

*B. dracunculifolia* is also known as a “benefactor” plant facilitating the development of other plant species (“beneficiary”) (Siqueira et al., 2022), in addition to improving the conditions of the habitat in which it is present (for example, humidity temperature, and availability of soil organic matter) (Lu et al., 2018). In that regard, Siqueira et al. (2022) evaluated the effects of *B. dracunculifolia* cultivation on the native plant community of an Atlantic Forest degraded area in a short period. Besides that, the study evaluated two areas of abandoned pasture colonized by grasses of the genus *Urochloa* spp, which originally represented riparian forest fragments in the Piranga River basin, one of the tributaries of the Rio Doce, in Minas Gerais, Brazil. In one of the areas, *B. dracunculifolia* was planted (restoration treatment); in the other, the pre-existing vegetation was maintained, remaining the same without intervention (degrading treatment). After 18 months of planting, the authors recorded all plant species (except *Baccharis* and grasses) and classified them as native, ruderal and invasive. The area cultivated with *B. dracunculifolia* (restored environment) showed greater species richness than the area without intervention (degraded environment). The values observed for species diversity in the areas exposed to this treatment were also higher with 17 exclusive native species, against three non-exclusive native species, in the treatment without intervention (degraded). Furthermore, fewer ruderal and invasive species were found in the areas restored with *B. dracunculifolia*, evidencing its importance in the recomposition of the native plant community, associated with the reduced chance of invasion by exotic species, and the possibility of use in the restoration of riparian forests, protecting mainly river springs (Siqueira et al., 2022).

#### 2.1.1 Synonymies

*Baccharis dracunculifolia* DC. includes the subspecies *Baccharis dracunculifolia* subsp. *Dracunculifolia* and *Baccharis dracunculifolia* subsp. *tandilensis* (Speg.) Giuliano. *Baccharis dracunculifolia* subsp. *Dracunculifolia* has six synonyms: *Baccharis bracteata* Hook. & Arn, *Baccharis dracunculifolia* f. *subviscosa* Kuntze, *Baccharis dracunculifolia* var. *integerrima* Kuntze, Kuntze, *Baccharis leptospermoides* DC., *Baccharis paucidentata* Sch. Bip. ex Baker and *Baccharis pulverulenta* Klatt (POWO Plants of the World Online, 2022). *Baccharis dracunculifolia* subsp. *tandilensis* has one synonym: *Baccharis tandilensis* Speg. (POWO Plants of the World Online, 2022).

### 2.1.2 Popular names

*B*. *dracunculifolia* has different popular names in the various regions in which it is found. In Brazil, it is popularly known as “alecrim-do-campo”, “alecrim-bassoura”, “vassoura”, “vassourinha”, “vassoureira”, “vassourão", “bassoura-branca” and “erva-de-São-João-Maria” (Viera, 2011; Alves et al., 2018; Casagrande et al., 2018; Bonin et al., 2020; Minteguiaga et al., 2022). The Guarani indigenous people call it *Ju’i vatã* (Pereira et al., 2016).

#### 2.1.3 Species description

**FIGURE 1:**
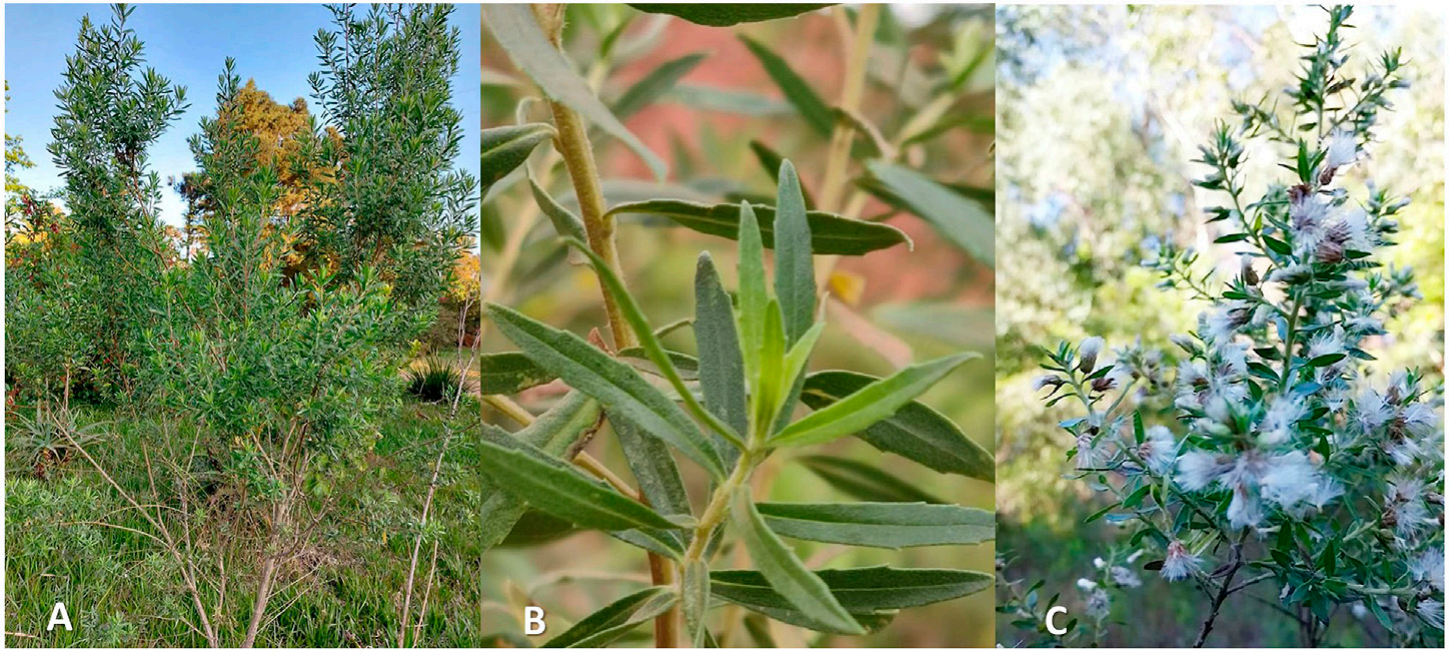
Adult specimen **(A)**, leaves **(B)**, and flowers **(C)** of *Baccharis dracunculifolia* DC. Source: The Authors.

*B*. *dracunculifolia* is a bush species that can reach 2.0–3.0 m in height, with hairy branches (Figure 1A) (Vieira, 2011). The leaves are lanceolate, membranous, uninerve, measuring 1.0–2.5 cm long and 3–4 mm wide, densely punctuated with glands, with entire margins or with one to three teeth, rarely with more than three teeth (Figure 1B) (Vieira, 2011). Regarding its flowers, this plant is multi-flowered, measuring between 3 and 4 mm in height and between 3 and 4 mm in diameter. The female flower has a corolla about 2 mm long and has a toothed edge, the male flower, pentasecta, is about 2.5 mm long, and the glabrous achene is approximately 1.5 mm long (Figure 1C) (Vieira, 2011).

#### 2.1.4 Geographic distribution

**FIGURE 2:**
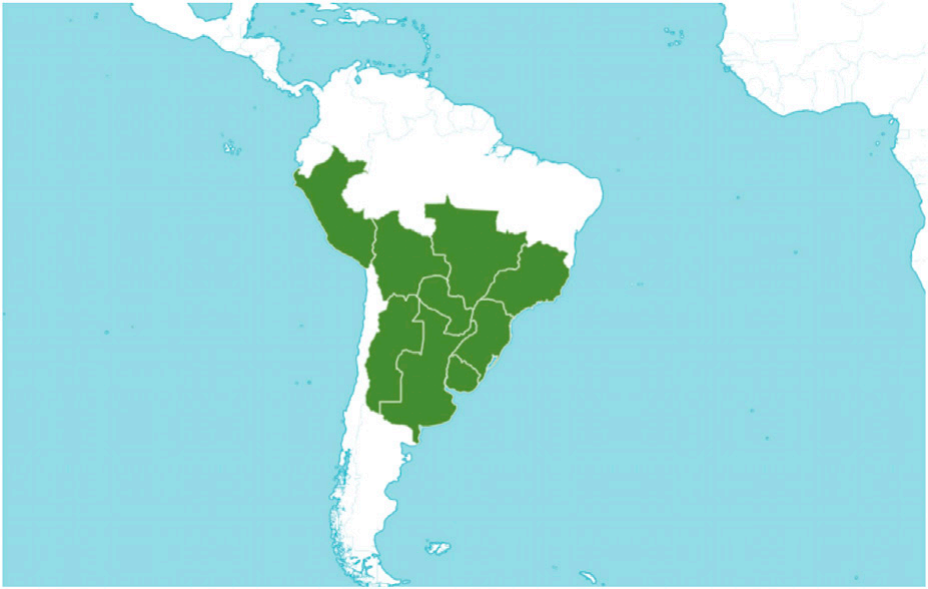
Geographical distribution of *Baccharis dracunculifolia* (Source: Plants of the World Online, Royal Botanic Gardens, Kew. 2022. Licensed under Creative Commons Attribution CC BY).

*B. dracunculifolia* is distributed in South America: Argentina (Northeast and Northwest), Bolivia, Brazil (South, Southeast, and West-Central), Paraguay, Peru, and Uruguay (Powo Plants of the World Online, 2022) (Figure 2). It is present in different types of vegetation, occurring both in the fields of the plateau and in the coastal sandbanks. Currently, its greatest occurrence is in anthropic areas: *capoeiras*, edges of forests and paths, clearings within capoeirões, and margins of swamps, making it one of the most characteristic Brazilian *Baccharis* as anthropic pioneers (Vieira, 2011). However, its phytogeographic domains are the Atlantic Forest, the Cerrado, and the Pampas (Heiden, 2022; Minteguiaga et al., 2022).

#### 2.1.5 Ethnobotanical uses

In traditional communities in Latin America, mainly in Argentina, Brazil and Bolivia, species of *Baccharis* L. are known and widely used. Traditional literature describes the use of these species as food, fodder for livestock, tools, and dyeing plants (Ritter et al., 2021).

The ethnobotanical knowledge that we have about the species that make up the genus Baccharis is directly related to their medicinal use. For example, in South American folk medicine, several species of Baccharis are used to treat malaria and parasitic infections (Parreira et al., 2010). These species are also indicated as antiseptics for the treatment of wounds and skin ulcers, as well as for treating fever and gastrointestinal diseases; they also act as spasmolytics, diuretics, analgesics and in the treatment of diabetes and infections of bacterial and fungal origin (Abad and Bermejo, 2007).

Due to its bush size and the greater strength of its wood (Figure 1A), in several Brazilian regions *B. dracunculifolia* is used in the manufacture of domestic and agricultural utensils and also as free wood. It is known as “vassoura” because it is used to build rustic brooms to sweep the floor mainly from outdoor areas. Another interesting aspect of this species is its use in religious rituals, mainly in communities in the states of Bahia and São Paulo, Brazil (Ritter et al., 2021).

Teas in the form of infusion and decoction prepared with the flowering plant (Figure 1C) are used in folk medicine to treat inflammatory processes, liver disorders and stomach ulcers (Salazar et al., 2018). It is an aromatic species used by the Guarani Indians in personal hygiene (Pereira et al., 2016).

## 3 Chemo-profiling

*B. dracunculifolia* is one of the most studied species from the phytochemical and pharmacological point of view within the genus *Baccharis*. The presence of phenolic acids, diterpenes, flavonoids, triterpenes, different types of glycosides and essential oils was shown when chemical evaluation of both aerial and underground parts was performed (Campos et al., 2016).

### 3.1 B. dracunculifolia essential oil

The economic importance of the essential oil composition of *B. dracunculifolia* (“vassoura” oil) has been widely described in the literature. The main compound, considered an important chemomarker, present in male and female individuals, is the acyclic alcohol sesquiterpene (E)-nerolidol, a compound with antiulcerative activity (Minteguiaga et al., 2021).

#### 3.1.1 Physical-chemical characteristics

**FIGURE 3:**
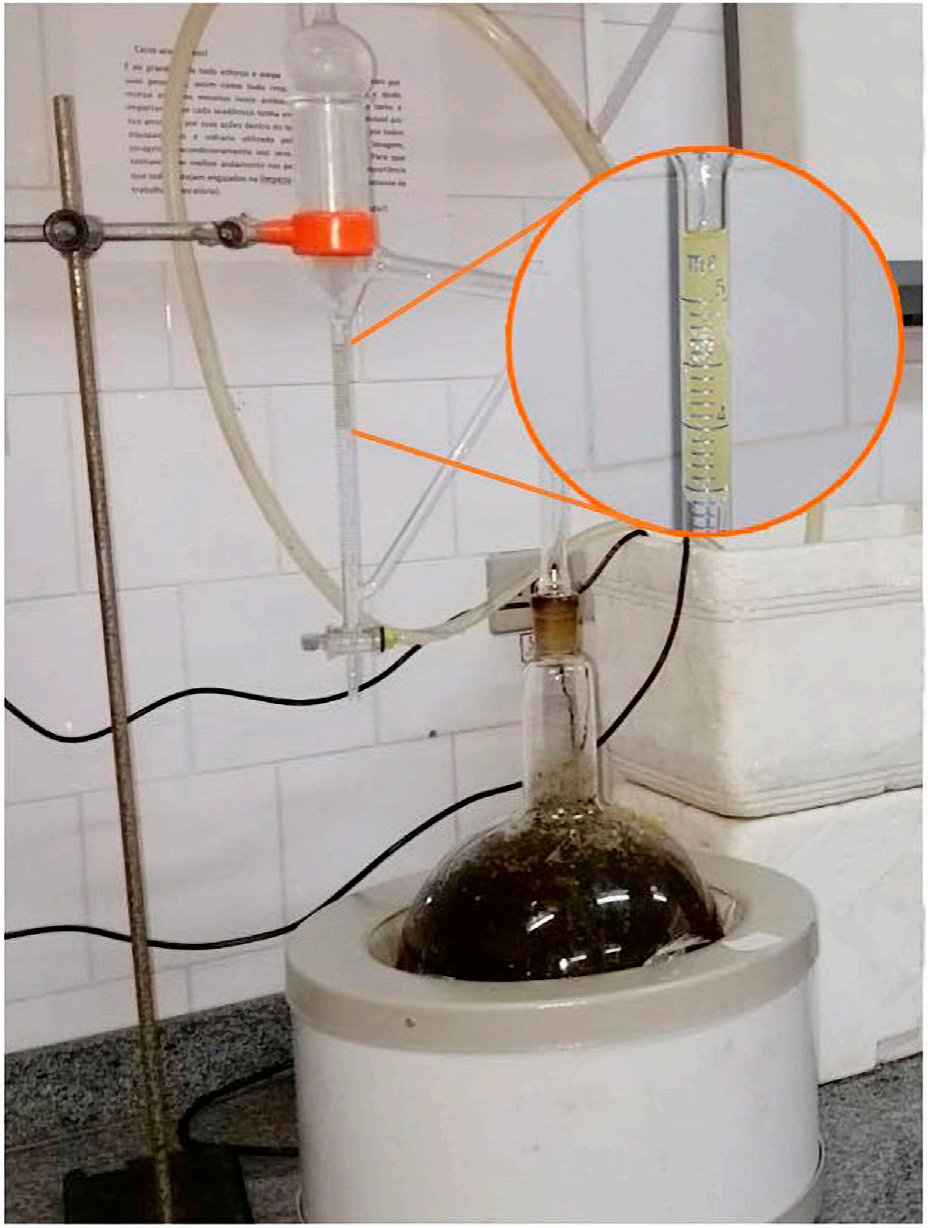
Essential oil obtained by hydrodistillation process of *B. dracunculifolia* leaves and flowers - Source: Chemical Laboratory of Natural Products-Paranaense University-UNIPAR, Brazil. Source: The Authors.

The essential oil is found in the aerial parts that comprise the leaves and flowers is colorless (Figure 3), with a characteristic honey odor (Cazella et al., 2019). The quality of essential oils is evaluated by physicochemical analyses. Some of the parameters to detect adulterations in essential oils are the determination of rotating power density and refractive index (Gil, 2007). To the product application and commercialization are necessary carrying out biological tests such as determining the yield of essential oils within the plant. The European Pharmacopoeia standardizes that 2 ml/kg is the minimum extraction yield of essential oils for product development and application (European Pharmacopoeia, 2013). The physicochemical indices of *B. dracunculifolia* essential oil are shown in Table 1.

**TABLE 1:** Physico-chemical indexes of *Baccharis dracunculifolia* essential oil.

Localization culture	Plant part	Physico-chemical indices				References
		Refraction index $n_{D}^{20}$	Specific rotation ${\left|\alpha \right|}_{D}^{20}$	Density (g/ml) $d_{20}^{20}$	Yield (%)	
Guaraniaçu, Paraná state, Brazil	Leaves and Flowers	1.4970	+23.63 ± 0.455	1.01 ± 0.001	1.8 ± 0.07	Cazella et al. (2019)
Francisco Beltrão, Paraná state, Brazil	Dry leaves	1.4593 ± 0.0005	+1.99 ± 0.06	0.9151 ± 0.0021	1.54	Fabiane et al. (2008)
Campos do Jordão, São Paulo State, Brazil	Fresh leaves	-	-	-	0.08 to 0.21	Lago et al. (2008)
Private Reserve of Natural Heritage Butuguara, Paraná state, Brazil	Aerial parts fresh and dried	-	-	-	0.31 and 0.43	Salazar et al. (2018)
Santa Helena, Paraná state, Brazil	Dry leaves	-	-	-	1.28	Brandenburg et al. (2020)
Franca, São Paulo state, Brazil	Dry leaves	-	-	-	0.60	Parreira et al. (2010)

(-): not reported by authors.

#### 3.1.2 Chemical composition

**FIGURE 4:**
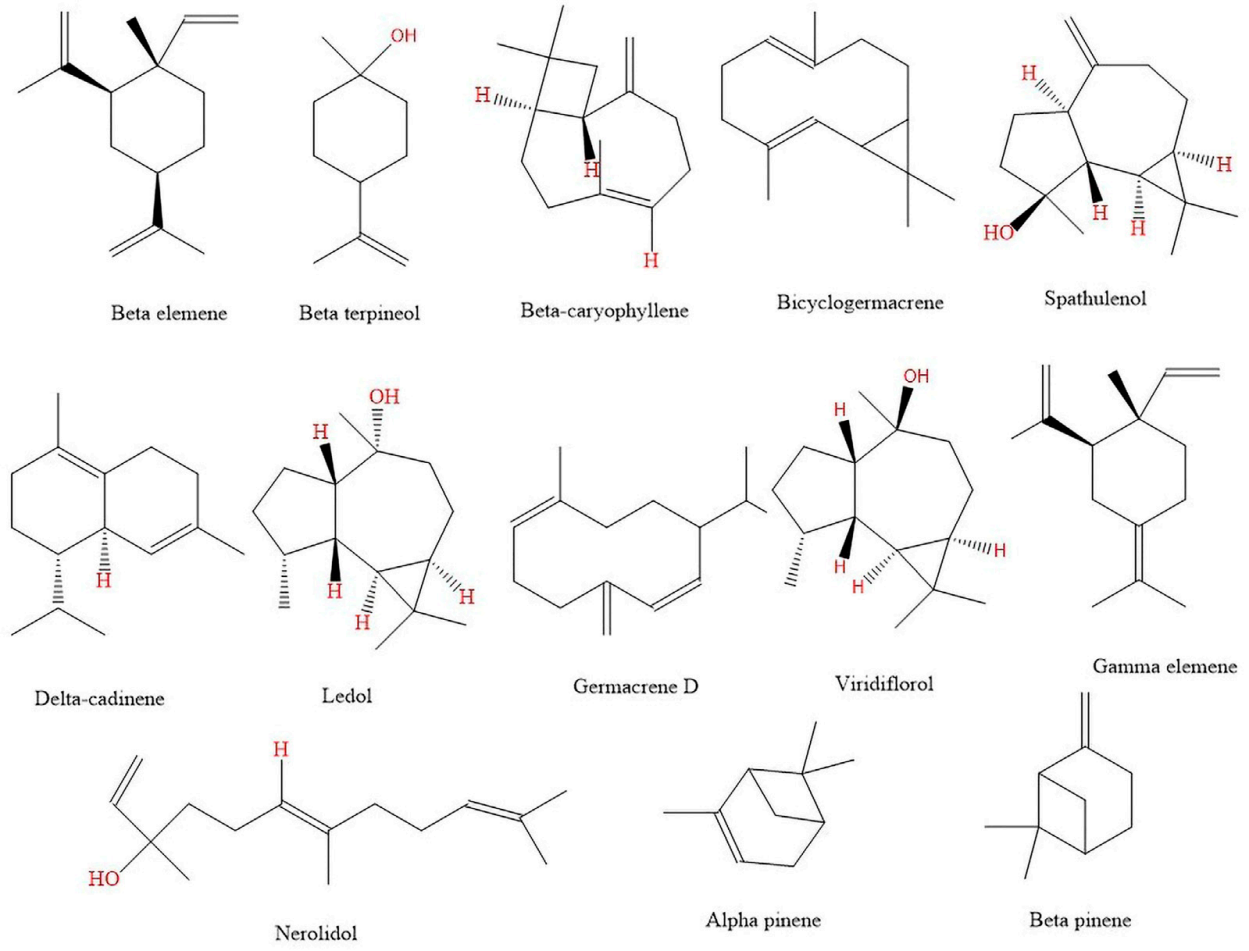
Chemical structures of majority volatiles constituents isolated from *Baccharis dracunculifolia*.

The presence of a complex structure of terpenoids was confirmed in the essential oil of *B. dracunculifolia*. Oxygenated sesquiterpenes nerolidol, sphatulenol, and the hydrocarbon sesquiterpenes germacrene D and byciclogermacrene are the major compounds (Cazella et al., 2019; Minteguiaga et al., 2022). However, factors such as the location of the crop implantation, climate, altitude, soil type, and especially the collection time can alter the concentration of these terpenoids. Thus, Table 2 and Figure 4 represent the chemical composition of the essential oil of *B. dracunculifolia* cultivated in different locations.

**TABLE 2:** Chemical Composition of *Baccharis dracunculifolia* essential oil.

Localization	Parts	Extraction and analysis technique	Essential oil chemical composition			References
Barra Bonita, São Paulo State, Brazil		Essential oil obtained from Dierberger S/A	The predominant class was oxygenated sesquiterpenes (86%). The major compounds were nerolidol (12.29%), spathulenol (5.12%), δ-cadinene (4.91%), β-caryophyllene (4.75%) e β-terpineol (4.03%)			Queiroga et al. (1990)
Campos do Jordão, São Paulo State, Brazil	Fresh leaves	Hydrodistillation (4 h). Analysis by GC/MS	The predominant class was hydrocarbon sesquiterpenes (63.10%), and the major compound was β-elemene (53.31%)			Lago et al. (2008)
Francisco Beltrão, Paraná State, Brazil	Dry leaves	Hydrodistillation (3 h). Analysis by GC/MS	The predominant class was monoterpenes 48.42% and sesquiterpenes (45.01%). The major compounds were β-pinene (27.45%); nerolidol (14.02); spathulenol (9.54%)			Fabiane et al. (2008)
Campinas, São Paulo State, Brazil	Aerial fresh parts	Steam distillation (1 h). Analysis by GC/MS	limonene (26.0%), α-pinene (10.8%), β-pinene (15.1%) and (E)-nerolidol (10.2%)			Queiroga et al. (2008)
Campinas, São Paulo State, Brazil	Aerial parts fresh	Steam distillation (2 h). Analysis by GC/MS	limonene (12.7%), germacrene D (9.8%), bicyclogermacrene (11.3%) and (E)-nerolidol (20.3%)			Queiroga et al. (2008)
Las Brujas, Uruguay	Aerial dry parts	Hydrodistillation. Analysis by GC/MS	β-pinene (27.7%), viridiflorol (11.2%), α-pinene (5.2), limonene (5.2%), spathulenol (7.5%), bicyclogermacrene (4.2%)			Frizzo et al. (2008)
Campestre da Serra, Rio Grande do Sul State, Brazil	Aerial dry parts	Hydrodistillation. Analysis by GC/MS	β-pinene (20.5%), limonene (13.1%), (E)-nerolidol (13.3%), spathulenol (11%), α-pinene (4.3%)			Frizzo et al. (2008)
Franca, São Paulo State, Brazil	Leaves	Hydrodistillation. Analysis by GC/MS	nerolidol (23.58%), germacrene-D (21.54%), bicyclogermacrene (19.24%), trans-caryophyllene (7.12%) and spathulenol (6.03%)			Massignani et al. (2009)
Franca, São Paulo State, Brazil	Dry leaves	Hydrodistillation. Analysis by GC/MS	germacrene D (2.18%), β-caryophyllene (2.28%), bicyclogermacrene (3.42%), d-cadinene (3.66%), a-muurolol (4.66%), spathulenol (16.24%), and nerolidol (33.51%)			Parreira et al. (2010)
Erechim, Rio Grande do Sul State, Brazil	Dry leaves and stems	Hydrodistillation. Analysis by GC/MS	β-pineno (24.6%), germacrene (10.9%) and cadinene (16.6%)			Reichert JR et al. (2013)
Viçosa, Minas Gerais State, Brazil	Fresh aerial parts	Hydrodistillation (5 h). Analysis by GC/MS	The predominant classes were sesquiterpenes hydrocarbons (24.4%) and oxygenated (27.8%). The major compound were nerolidol (22.3%); germacrene-D (7.2%) and bicyclogermacrene (6.5%)			Lage et al. (2015)
Canelinha, Santa Catarina State, Brazil	Aerial parts	Steam-dragging distillation	β-pinene (9.94%), d-limonene (9.59%), β-nerolidol (7.93%), caryophyllene (7.69%), spathulenol (6.69), α-muurolene (6.74%) and α-pinene (5.31%)			Chaaban et al. (2018)
Private Reserve of Natural Heritage Butuguara, Paraná State, South Brazil	Aerial parts (dry and fresh)	Hydrodistillation (4 h). Analysis by GC/MS		The predominant classes were sesquiterpenes hydrocarbons (33.2; 15.5%) and oxygenated 45.7 (47.3%). The major compound were germacrene D (18.4; 5.0%); (E)-nerolidol (14.0; 8.2%); spathulenol (11.0; 12.0)	Salazar et al. (2018)	
Bento Gonçalves, Rio Grande do Sul State, Brazil	Dry leaves	Steam distillation (1 h). Analysis by GC/MS		β-pinene (18.01%), ledol (13.55%), spathulenol (13.43%) and limonene (10.11%)	Pedrotti et al. (2019)	
Guaraniaçu, Paraná state, Brazil	Aerial parts (dry leaves and flowers)	Hydrodistillation (2 h). Analysis by GC/MS		The predominant classes were oxygenated sesquiterpenes (60.8%) and hydrocarbon sesquiterpenes (22.9%). The major compounds were spathulenol (27.4%) and trans-nerolidol (23.1%)	Cazella et al. (2019)	
Santa Helena, Paraná State, Brazil	Dry leaves	Hydrodistillation (3 h). Analysis by GC/MS		limonene (6.76%), β-caryophyllene (8.44%), bicyclogermacrene (14.18%) and nerolidol (8.02%)	Brandenburg et al. (2020)	
Londrina, Paraná State, Brazil	Fresh leaves	Hydrodistillation (3 h). Analysis by GC/MS	cis-trans nerolidol (17.58%); γ-elemene (15.06%); d-limonene (10.54%); caryophyllene (9.76%); β-pinene (9.57%); spathulenol (8.49%)			Luchesi et al. (2022)

### 3.2 B. dracunculifolia leaf and flowers extracts

The main chemical constituents in plants of the genus *Baccharis* are phenolic and terpenoid compounds. In addition, diterpene and flavonoid compounds and phenolic acids such as derivatives of cinnamic and chlorogenic acids (Supplementary Figure S1) are also present. Diterpenes are among the most abundant compounds present in the species of this genus, highlighting those with neo-clerodane, labdane, and kaurane skeletons (Supplementary Figure S2) (Burgos et al., 2022).

In an experiment conducted by Nagatani et al. (2002a), using aerial parts of *B. dracunculifolia* collected in the city of São Paulo, Brazil. From this plant material, a methanolic extract was prepared and this was partitioned in water. The suspended fraction was then extracted with diethyl ether, resulting in two fractions: ethyl and aqueous. The ethyl fraction was concentrated and the residue partitioned into two solutions: the first composed of benzene-n-hexane (1:1) and the second composed of 80% methanol in water. The residue derived from the methanolic solution was separated on a silica gel column and analyzed by High Performance Liquid Chromatography (HPLC) indicating the presence of the flavonoids naringenin, acacetin, dihydrokaempferol, isosakuranetin, kaempferide, among others (Nagatani et al., 2002a) (Supplementary Figure S3).

According to Minteguiaga et al. (2022), the phenolic acids such as gallic, *p*-coumaric, ferulic (E)-cinnamic, hydroxycinnamic, caffeic, dihydrocoumaric, and several caffeoylquinic acids derivatives are common components when medium polar to polar organic and hydroalcoholic extracts of *B. dracunculifolia* are studied (Supplementary Figure S4).

**FIGURE 5:**
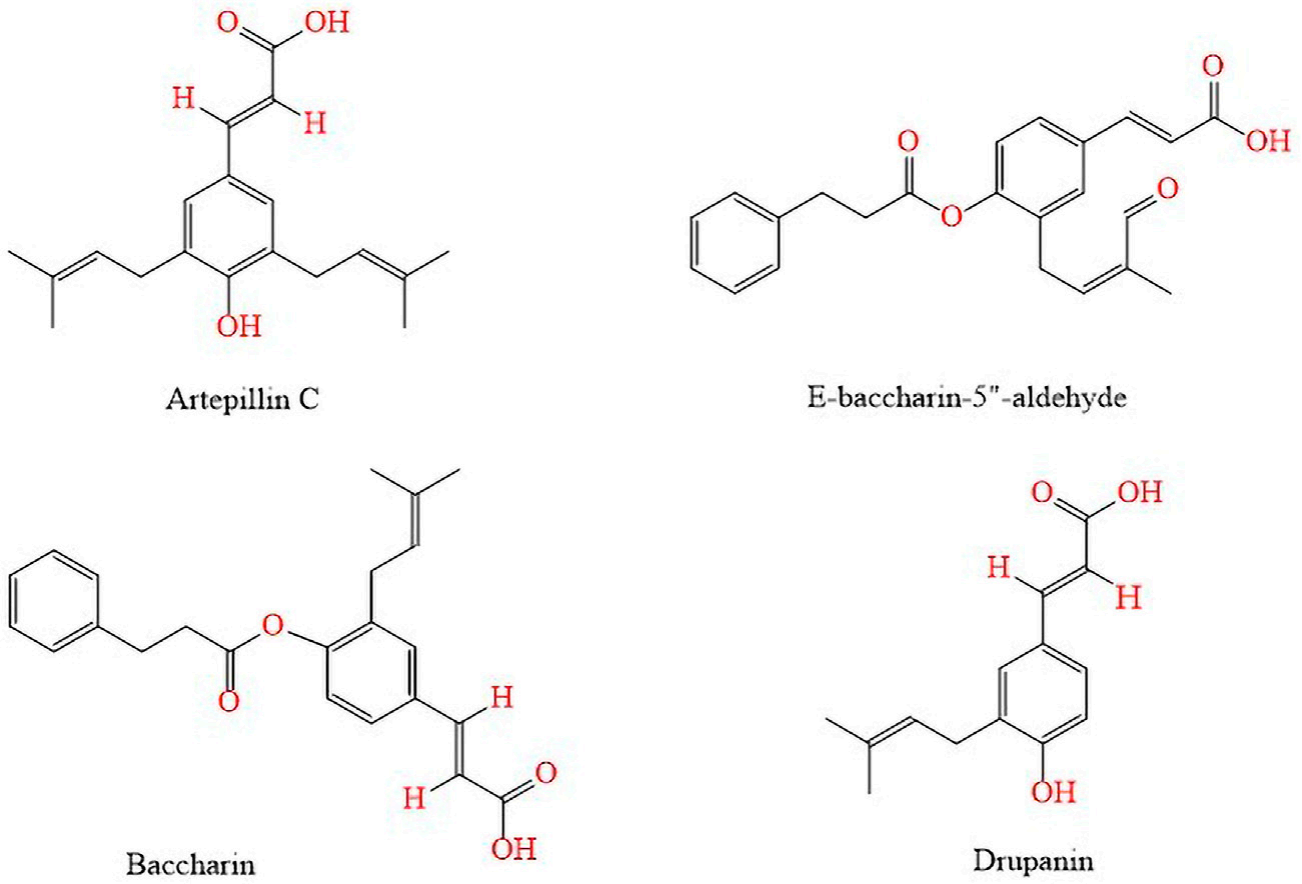
Prenylated derivatives and cinnamic derivatives found in *B. dracunculifolia* hydroalcoholic extracts.

The prenylated derivatives such as 3,5-diprenyl-*p*-coumaric (artepillin C), 3-prenyl-4-dihydrocinnamoiloxy-cinnamic (baccharin), baccharin-5″-aldehyde and 3-prenyl-*p*-coumaric (drupanin) are also frequents (Minteguiaga et al., 2022) (Figure 5).

**FIGURE 6:**
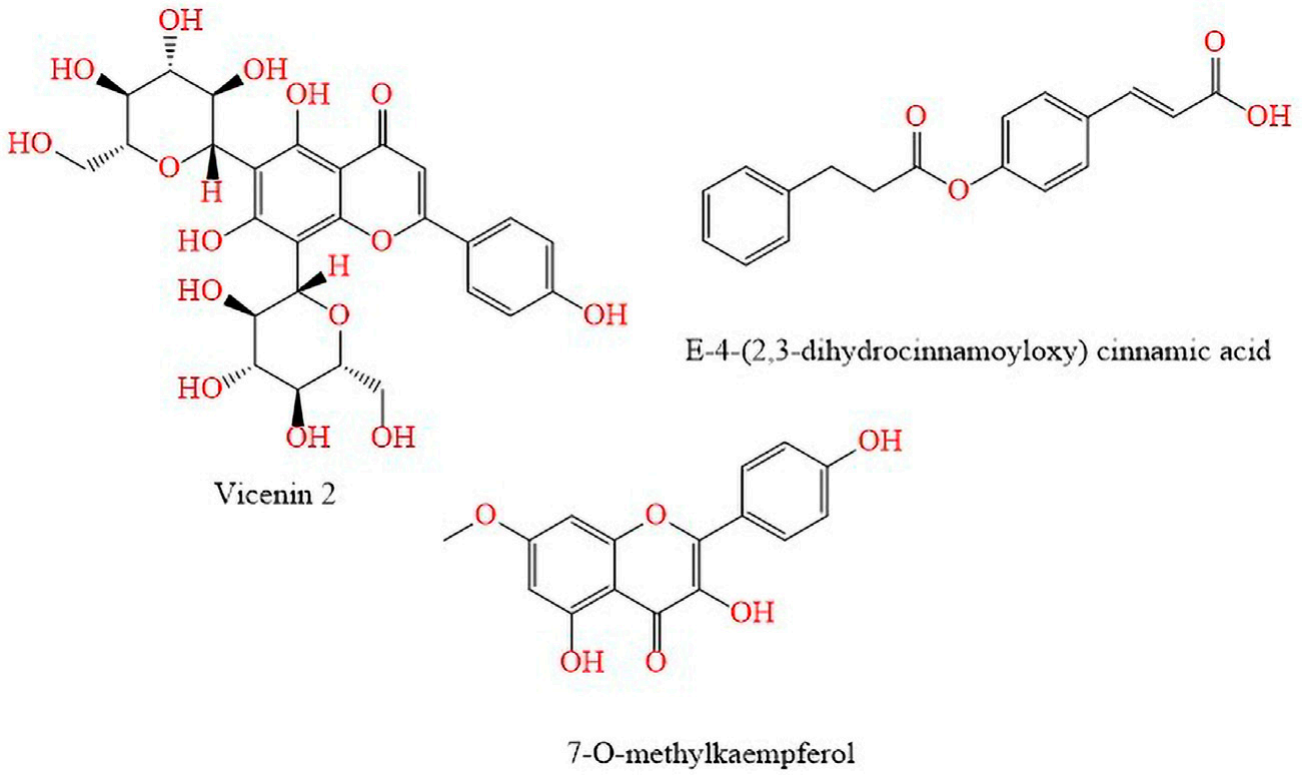
Flavonoids and phenolic acids found in the hydroalcoholic extract of *B. dracunculifolia* leaves and green propolis.

Other flavonoids and phenolic acids such as baccharin, *E*-baccharin 5″-aldehyde, vicenin, *p*-coumaric acid, 7-O-methylkaempferol and E-4-(2,3-dihydrocinnamoyloxy) cinnamic acid; present in the hydroalcoholic extract of *B. dracunculifolia* leaves and green propolis were isoleted and analyzed by HPLC-DAD (diode array detector) by Rodrigues et al. (2020) (Figure 6).

The hydroalcoholic extract of lyophilized *B. dracunculifolia* aerial parts was investigated by ultra-high-performance liquid chromatography–high-resolution mass spectrometry (UHPLC–HRMS) by Bonin et al. (2020). The flavonoids often reported as major constituents of green propolis are; naringenin, apigenin, kaempferol and kaempferide. The flavonoids pinobanksin, chrysin, apigenin, betuletol, galangin, pinocembrin, quercetin, were also identified in the hydroalcoholic extract of *B. dracunculifolia* (Bonin et al., 2020) (Supplementary Figure S5).

**FIGURE 7:**
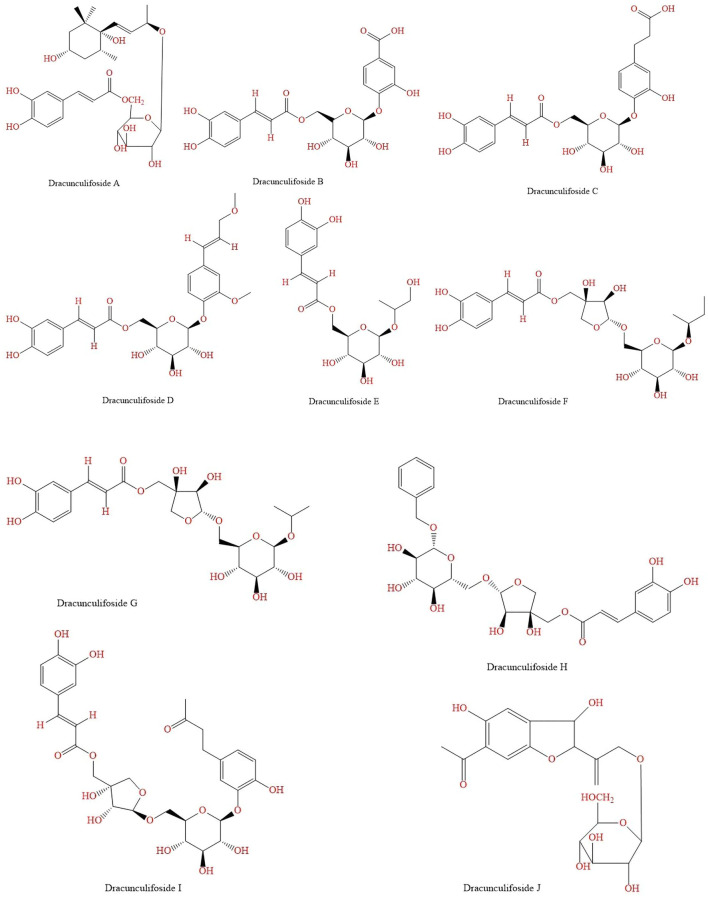
(Continued). **(A)** Glycosides found in *B. dracunculifolia* dried aerial parts methanolic extract. **(B)** Glycosides found in *B. dracunculifolia* dried aerial parts methanolic extract.

Ten new glycosides, called dracunculifosides A-J (Figure 7A and Figure 7B), were isolated from *B. dracunculifolia* by Nagatani et al. (2002b). Using the dried aerial parts, a methanolic extract was prepared and suspended in water. Then, with diethyl ether and partitioned into an ether-soluble fraction and a water-soluble fraction, the suspension was extracted. Through a Mitsubishi Diaion HP-20 column the water-soluble fraction was passed, then, the adsorbed material was subsequently eluted with 50% methanol in water, 70% methanol in water, and methanol 100%. The residue of 50% methanol eluate from the Diaion HP-20 column was concentrated was re-chromatographed on a silica gel column and by semi-preparative HPLC. The structures of these glycosides were determined on the basis of spectral and chemical evidence, beeing β-d-glucopyranose or β-d-apiofuranosyl-(1→6)-β-d-glucopyranose and most possess an (E)-caffeoyl group (Nagatani et al., 2002b).

**FIGURE 8:**
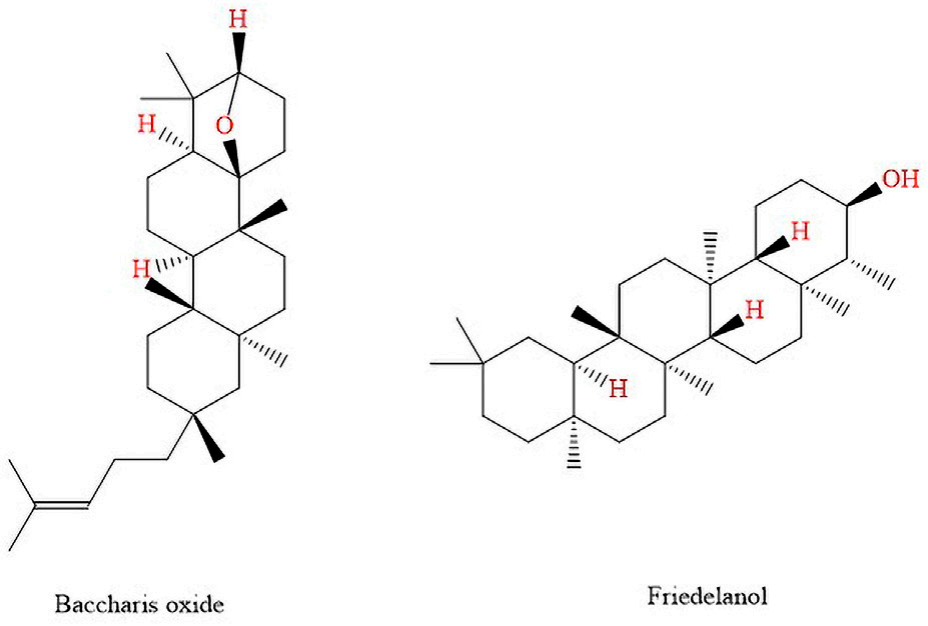
Pentacyclic triterpenoids found in *B. dracunculifolia* leaves and roots extract.

The chemical composition of crude extract obtained from the aerial parts of *B. dracunculifolia* was investigated by Missima et al. (2007). This extract, then, was dissolved in methanol:water (7:3) and submitted to a sequential partition with hexane and dichloromethane, being this last fraction, chromatographed over silica gel under a Vacuum-liquid chromatography system, using hexane–ethyl acetate mixtures in increasing proportions as eluent, resulting in five fractions. In the first fraction the compound *Baccharis* oxide was identified, and in the second fraction the compound friedelanol, two pentacyclic triterpenoids were found (Missima et al., 2007) (Figure 8).

**FIGURE 9:**
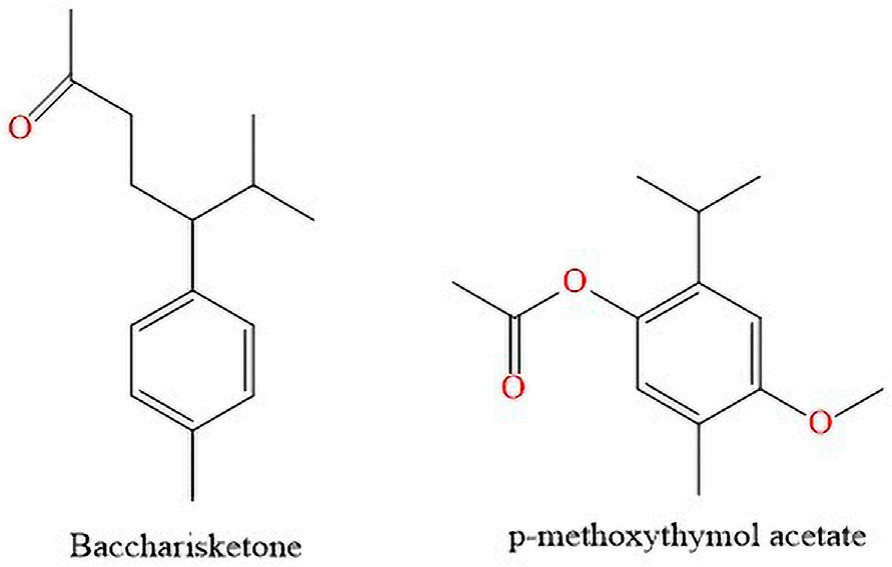
Monoterpenes found in *B. dracunculifolia* leaves extract.

Fukuda et al. (2006) isolated two new monoterpenes baccharisketone and *p*-methoxythymol acetate (Figure 9). Fukuda et al. (2006) isolated two new monoterpenes bacarisketone and p-methoxythymol acetate (Figure 9) from the extract of dried leaves of *B. dracunculifolia* using ethanol as extractant liquid. The extraction technique was by ultrasound-assisted extraction, the residue obtained was chromatographed in an HP-20 column and eluted successively with pure methyl alcohol at concentrations of 40% and 70% and 100% acetone. The methanolic fraction was separated on a silica gel column and eluted with n-hexane-ethyl acetate at different graduations. then the identification was performed by HPLC.

### 3.3 B. dracunculifolia green propolis

Propolis is a plant resin that bees take from plants. In general, propolis is composed of essential and aromatic oils, waxes, resins and balm, pollen and other substances. Depending on the geographic origin of propolis and the vegetation from which it is extracted, its color can vary from green, to red and dark brown (Ferreira and Negri, 2018). *B. dracunculifolia* propolis, also known as green propolis, is produced from the vegetative apices of this species. As it is a mixture of products dependent on several external factors, the chemical analysis of propolis is challenging, this analysis is performed mainly on aqueous and ethanolic extracts as they are the most used pharmaceutical forms in different types of therapeutic applications (Nascimento et al., 2008).

Samples of Brazilian green propolis from different locations were investigated for chemical composition. The samples were extracted with methyl alcohol by ultrasound-assisted technique (360 W, 25 KHz) for 30 min. Extracts were analyzed by UHPLC coupled with UHPLC-ESI-QTOF-MS. The compounds identified were: chlorogenic acid, caffeic acid, isochlorogenic acid A, isochlorogenic acid B, isochlorogenic acid C and artepillin C (Sun et al., 2019). Compounds identified in green propolis were also found in investigations carried out by Chuang et al. (2022) (Supplementary Figure S6A,B). To complement the chemical composition of green propolis, Ferreira and Negri (2018) described in their studies the presence of triterpenoids, such as α- and β-amyrins, and their respective acetates. Chen et al. (2008) complemented the identification indicating the presence of the triterpene pentacyclic baurenyl acetate, the mono and di-caffeoylquinic acids and the flavonoids kaempferol and luteolin (Supplementary Figure S6A,B).

## 4 Pharmacology and bioactivity

### 4.1 Anti-inflammatory

#### 4.1.1 Essential oil

**FIGURE 10:**
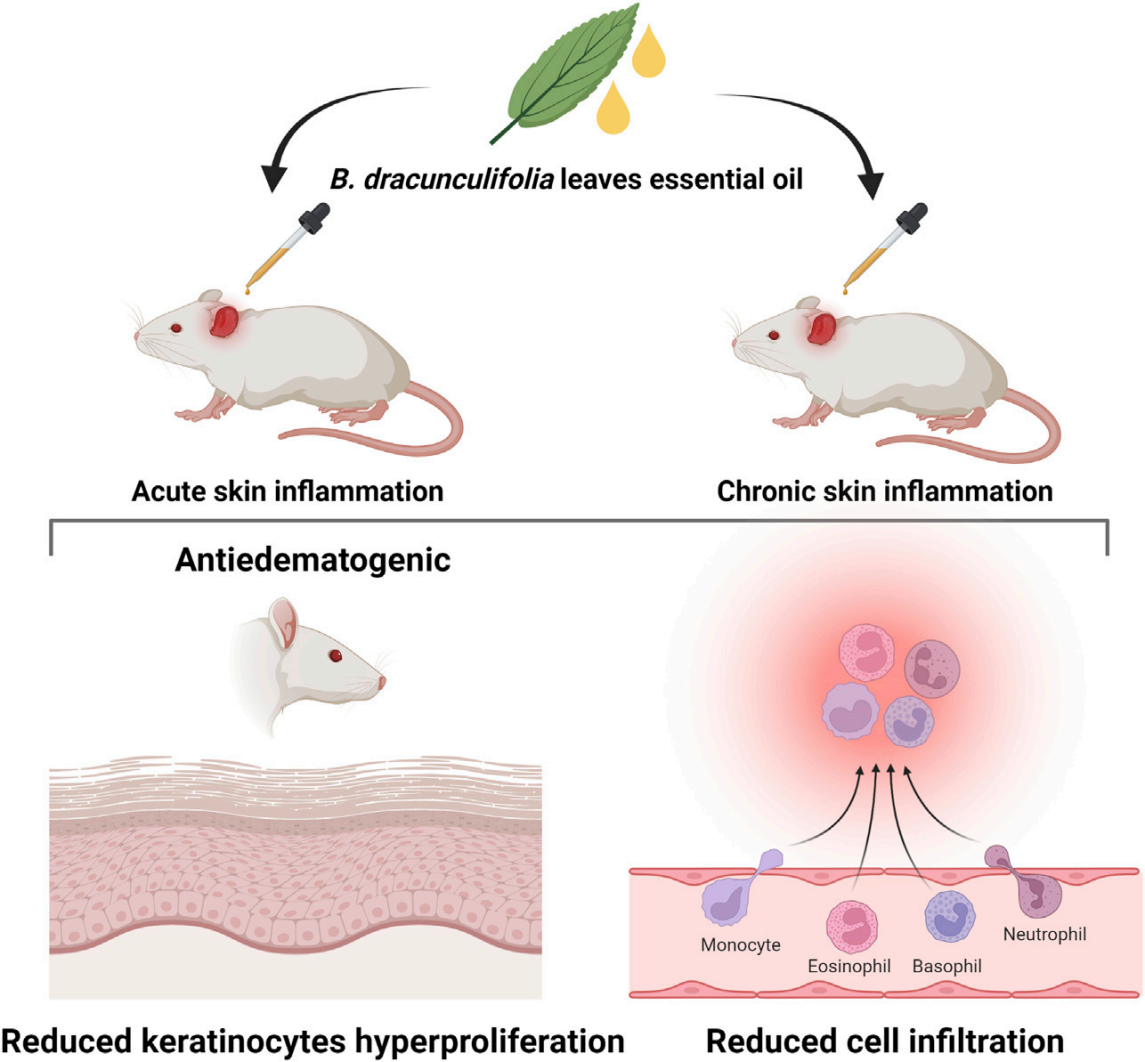
Anti-inflammatory activity of *B. dracunculifolia* leaves essential oil evaluated in models of skin inflammation (Brandenburg et al., 2020).

The anti-inflammatory performance of *B. dracunculifolia* leaves essential oil was evaluated in models of skin inflammation by Brandenburg et al. (2020) (Figure 10). Essential oil doses were applied to the ear of mice where acute skin dermatitis was previously induced by 12-O-tetradecanoylphorbol-acetate (TPA) or arachidonic acid (AA). The topical application of 0.1 and 1.0 mg/ear essential oil reduced the TPA-induced edema by 67.4% and 81.8% after 6 h and by 85.6% and 95.0% after 24 h, respectively. The essential oil also reduced the leucocytes migration by 91.6% (1.0 mg/ear) and cell infiltrate by 97.1% (1.0 mg/ear). The use of 1.0 mg per ear of essential oil reduced the edema in the AA-induced dermatitis by 55%. In both models of acute inflammation, the oil showed an antiedematogenic effect similar to the positive controls dexamethasone (TPA-model) and indomethacin (AA-model). In the chronic inflammation model, multiple applications of TPA were used to induce ear skin inflammation. The essential oil (1.0 mg/ear) was topically applied twice a day from the fifth to the ninth day. At the end of the treatment, the essential oil inhibited all inflammatory parameters evaluated as ear edema (61.4%), reduced the inflammatory cell influx (74.1%), *epidermis* thickness (85%), and keratinocyte proliferation (74.3%), and differentiation (82%). The results of Brandenburg et al. (2020) highlight, therefore, the anti-inflammatory potential of the essential oil of *B. dracunculifolia* that shown to act at different points of the inflammation with good anti-inflammatory efficacy. However, in the absence of local or systemic side effects. Additionally, the authors (Brandenburg et al., 2020) suggest that the anti-inflammatory and antiproliferative activities observed might be due to the sesquiterpenes present in the essential oil (Figure 4).

#### 4.1.2 Extract

The anti-inflammatory potential of ethyl acetate extract from aerial parts of *B. dracunculifolia* was evaluated by Cestari et al. (2011) in a model of colitis *in vivo*. Tests of acute and chronic colitis were conducted. In the acute colitis model, rats orally received plant extract in concentrations of 5–200 mg/kg per day before colitis induction and 24 h after that. The doses of 5 and 50 mg/kg macroscopically reduced the damage score of the lesions (de 8.5 para 6.0 e 6.5, respectively, on a scale of 0–10) in the acute phase of the inflammatory process. *B. dracunculifolia* 5 mg/kg extract could counteract the colonic glutathione (GSH) depletion resulting from the colonic oxidative damage induced by trinitrobenzenesulfonic acid. Also, the myeloperoxidase (MPO) and alkaline phosphatase (AP) activities, commonly used as inflammatory markers, were reduced in animals treated with the extract. In the chronic colitis model, the extract reduced damage to the colon and the enzymatic activity of biochemical markers of inflammation after the first week. Cestari et al. (2011) suggest that the anti-inflammatory activity was related to the phenolic compounds as caffeic and *p*-coumaric acids (Figure 8), drupanin, baccharin, mainly artepillin C (Figure 5), the major component.

Figueiredo-Rinhel et al. (2019) used the ethanolic extract from *B. dracunculifolia* leaves and caffeic acid (Supplementary Figure S4) to examine its anti-inflammatory action *in vivo*. The authors tested the effects of free caffeic acid and extract and these compounds included in liposomes to modulate inflammation *in vitro* and *in vivo*. Liposomal extract and caffeic acid inhibited the neutrophil superoxide anion and total reactive oxygen species production. In the *in vivo* inflammation model, the free extract reduced edema, cell infiltration, and synovial levels of cytokines. The encapsulated extract also presented an anti-inflammatory effect *in vivo*, but with an effective dose lower. The same occurred when encapsulated caffeic acid was used, with an effective dose reduction of about sixteen times. The results demonstrate that biocompatible liposomes improve the *B. dracunculifolia* extract anti-inflammatory action *in vivo* (Figueiredo-Rinhel et al., 2019).

Lima et al. (2020) evaluated the efficacy of the ethanolic extract of *B. dracunculifolia* leaves in treating diversion colitis, a complication of intestinal transit diversion. Rats colostomy patients were treated for 21 days with an intrarectal infusion of *B. dracunculifolia* extract that reduced inflammation (from 2.7 to 2.1 on a scale ranging from 0 to 3). Still, they did not change vascular congestion in the excluded segment (Lima et al., 2020).

In a recent research conducted by França et al. (2022) the hydroalcoholic extract of *B. dracunculifolia* and isolated *p*-coumaric acid (Supplementary Figure S4) were used to treat colitis *in vivo*. Treatment with the extract at 300 mg/kg caused a 39.2% reduction in the disease activity index (DAI) score, prevented weight loss, preserved some histological features, and increased mucin by 318%, evidencing the pharmacological potential of the extract to treat inflammatory bowel disease. However, *p*-coumaric acid alone could not attenuate DAI, suggesting that the intestinal anti-inflammatory effects observed were due to the combination of extract constituents (França et al., 2022).

Due to the relationship between inflammatory bowel disease and neuropsychiatric comorbidities, França et al. (2022) also evaluated the neuroprotective action of the hydroalcoholic extract of *B. dracunculifolia* in mice with colitis. In different tests to measure anxious and depressive behavior, mice with colitis treated with the extract exhibited behavior similar to the healthy animals. These effects may have resulted from attenuation of central nervous system inflammation due to the preservation of intestinal barrier integrity or the inhibitory effects on inflammatory markers caused by the extract (França et al., 2022).

#### 4.1.3 Green propolis

The effect of green propolis extract was investigated by Hori et al. (2013). In their studies, the researchers related the potential of green propolis in reducing the secretion of interleukin one beta (IL-1β) in mouse macrophages and verified that extract (30 μg/ml) did not show toxicity in the cells. This effect can be associated with the compounds found in Brazilian green propolis extract, such artepillin C, that plays a role in regulating inflammasomes (Hori et al., 2013).

In a model of dextran sulfate sodium-induced colitis in mice, Mariano et al. (2018) evaluated the anti-inflammatory potential of oral administration of hydroalcoholic extract of green propolis for 7 days (3, 30 and 300 mg/kg). The treatment with 300 mg/kg of green propolis extract reduced macroscopic and mucosal damage in the colon and reversed the decrease in mucin levels. Other effects were the recovery of the superoxide dismutase (SOD) activity and the levels of GSH in the colon. The authors suggest that the maintenance of the intestinal mucin and the activation of antioxidant system defense had a central role in the green propolis anti-inflammatory effect. However, green propolis extract did not change the patterns of gastric (emptying) and intestinal (transit rate) functioning of mice (Mariano et al., 2018).

Wang et al. (2018) compared the anti-inflammatory effects of Brazilian *Baccharis* green propolis and Chinese poplar-type propolis in animal models of colitis. The polyphenolic composition of the two propolis showed to be different. Of the 27 compounds identified, only five were detected in the Brazilian propolis, with artepillin C (Supplementary Figure S6A) the most abundant. On the other hand, 20 compounds were identified in Chinese propolis rich in caffeic acid phenethyl ester (CAPE), but artepillin C was not detected. The animals were orally treated with 300 mg/kg of Brazilian and Chinese propolis with an initial dose 1 week before treatment with DSS for 1 week, followed by 3 days without DSS. Both propolis significantly reduced the disease activity index, preventing damage to colonic tissue, reducing malonaldehyde levels, and increasing antioxidant capability, indicating greater resistance to DSS-induced colonic oxidative stress. The two propolis also diminished apoptosis in the colon and suppressed the inflammation markers IL-1β, IL-6, and monocyte chemoattractant protein-1 (MCP-1). However, only Brazilian propolis induced the expression of transforming growth factor β. Only Chinese propolis raised the diversity of intestinal microbiota. Wang et al. (2018) concluded that the two propolis demonstrated similar anti-inflammatory capacity despite some physiological impacts and different compositions.

Miranda et al. (2019) analyzed the anti-inflammatory potential of green propolis extract in a chronic inflammation model in mice submitted to a low-protein diet. Animals received diets with 12% (standard) or 3% (low) protein. After 28 days of starting the diet, inflammation was induced, and the treatment of daily doses of 500 mg/kg of the extract was initiated until seven or 15 days. The treatment with the extract stimulated weight restoration and conservation of serum protein levels in animals on a diet restricted in proteins. It also selectively altered the hematological parameters, increasing leukocyte recruitment. Additionally, the extract had a modulatory action in the inflammatory kinetics that was less efficient in the groups in a low-protein diet (Miranda et al., 2019).

Xu et al. (2020) tested the protective effect of green propolis extract using an *in vitro* model of lipopolysaccharide-stimulated mouse aortic endothelial cells (MAECs). All concentrations (5, 10, and 20 μg/ml) of extract increased cell survival, and the 20 μg/ml dose inhibited the expression of inflammatory cytokines and reduced adhesion molecules. In addition, the authors detected prenylated derivatives of *p*-coumaric acid, diterpenes, and flavonoids in the extract. Artepillin C (35.68%) was the major compound, followed by kaempferide (7.06%), 3-isoprenyl p-coumaric acid (6.19%), pinocembrin (5.56%), diisoprenyl-p-coumaric acid isomer (4.49%), 40-methoxy pinobanksin (4.10%) and 3-hydroxy-2,2-dimethyl-8-prenylchromane-6-propenoic (3.39%) (Xu et al., 2020) (Supplementary Figures S6A,B).

Yuan et al. (2020) also compared the anti-inflammatory action of Brazilian *Baccharis* propolis and Chinese poplar propolis in a murine model of systemic inflammation induced by lipopolysaccharide (LPS). Brazilian and Chinese propolis ethanol extracts (0.1 ml) were administered intragastrically in mice on the day before LPS administration, 2 hours before LPS administration, and 12 h after LPS administration. Both propolis showed similar anti-inflammatory activity, evidenced by the reduction of genic expression and reversion of the serum cytokines levels in the animals that received prophylactic doses of propolis extract. The authors unveiled differences between the propolis chemical profiles with much higher content of artepillin C, chlorogenic acid, and isochlorogenic acid A (Supplementary Figure S6A) in the Brazilian propolis and great CAPE levels and pinocembrin in Chinese propolis that could contribute to the anti-inflammatory effect (Yuan et al., 2020).

Ferreira et al. (2021) evaluated the actions of *p*-coumaric acid and baccharin (500 or 1,000 μg/kg) isolated from green propolis in a murine air pouch LPS-induced inflammation model. The two doses of *p*-coumaric acid and baccharin (Supplementary Figures S4, S5) had similar effects in reducing leukocytes and neutrophil recruitment. Additionally, histological analysis showed that the accumulation of neutrophils induced by LPS was significantly reduced by treatment with 500 μg/kg *p*-coumaric acid and baccharin. The author also reported significant reductions in nitric oxide (NO) production and protein extravasation in inflamed air pouches treated with 500 μg/kg *p*-coumaric acid or baccharin, indicating a role in vascular barrier preservation of these compounds. The evaluation of inflammatory markers showed that baccharin reduced IL-6, TNF-α, and IL-1β levels, while *p*-coumaric acid reduced TNF-α and IL-1β levels. However, *p*-coumaric acid stimulated interleukin 10 (IL-10) production. Treatment with *p*-coumaric acid or baccharin also significantly impacted levels of the eicosanoids prostaglandin F2α (PGF2α), 15-hydroxyeicosatetraenoic acid (15-HETE), and 12-HETE, but only *p*-coumaric acid suppressed 5-HETE synthesis. Apparently, *p*-coumaric acid and baccharin had different molecular mechanisms to modify the inflammatory response (Ferreira et al., 2021).

In a recent systematic review, Soleimani et al. (2021) analyzed eight studies on the anti-inflammatory action of green propolis on various aspects of inflammatory bowel disease in preclinical studies. Green propolis effectively improves histological inflammation aspects of the colon and clinical and morphological features in animals with colitis. The propolis benefit suggests a relationship between its protective effects against oxidative stress and endogenous antioxidant parameters. Soleimani et al. (2021) suggest that the likely mechanism of action includes impeding transcription factors and proteins.

### 4.2 Antiulcerogenic

#### 4.2.1 Essential oil

Klopell et al. (2007) evaluated the antiulcerogenic potential of essential oil from leaves of *B. dracunculifolia* in an acute gastric injury model. Oral administration of essential oil significantly decreased the rate of ulcerative wounds (42.79–61.61%) when compared to control (vehicle) and omeprazole (30 mg/kg). Essential oil analysis showed the presence of mono- and sesquiterpenes and the major compound nerolidol (Figure 4), which was used alone at concentrations (50, 250, and 500 mg/kg) in the evaluation of antiulcerogenic activity in rats. The two highest nerolidol concentrations significantly reduced the ulcerative lesion rate in all models evaluated, emphasizing the 87.6% reduction observed when 500 mg/kg of nerolidol was used in an ethanol-induced ulcer model (Klopell et al., 2007).

Massignani et al. (2009) used the essential oil from *B. dracunculifolia* leaves to treat rats wich had ulcers induced by non-steroidal anti-inflammatory drugs, ethanol, and stress. They observed that the lesions index, total area and the number of wounds were lower. These results added to a decrease in gastric juice volume and total acidity, and absence of toxicity (Massignani et al., 2009).

#### 4.2.2 Extract

Hydroalcoholic extract of *B. dracunculifolia* aerial parts decreased the rate of wounds in ulcer models induced by ethanol, indomethacin, and stress (Lemos et al., 2007). At the highest concentration, the extract caused inhibition from 75.6% (indometacin model) to 95% (ethanol model). Additionally, the extract reduced the stomach juice volume and increased pH in a gastric secretion evaluation model (Lemos et al., 2007).

Costa et al. (2019) stated that extract of *B. dracunculifolia* leaves (30, 100, and 300 mg/kg) demonstrated the ability to heal gastric ulcers in models of chronic ulcers induced by acetic acid. The extract oral administration decreased the ulcer area between 30% and 50% compared to vehicle. Additionally, the authors characterized and isolated the major compounds from the extract [ferulic acid (Figure 4), *p*-coumaric acid, caffeic acid (Supplementary Figure S4), artepillin C, baccharin (Figure 5), and aromadendrin-4′-O-methyl ether] to assess their gastric healing ulcer activity. *p*-coumaric acid reduced the ulcer area by 66%, while the other compounds showed no healing activity compared to the vehicle (Costa et al., 2019). After evaluating several physiological parameters, Costa et al. (2019) suggested that *B. dracunculifolia* extract recovers ulcerated gastric tissue, increasing mucus and antioxidant enzymes, and reducing gastric proton pump activity.

Recently Boeing et al. (2021) re-evaluated the effects of *p*-coumaric acid (10 mg/kg) extracted from *B. dracunculifolia* in rats with gastric ulcer. Oral *p*-coumaric acid reduced the extent of the ulcer base by 44.6%. It reduced the damage to the mucosa and submucosal layers compared to the vehicle, reaffirming the antiulcerogenic action of *p*-coumaric acid (Boeing et al., 2021).

#### 4.2.3 Green propolis

Barros et al. (2007) evaluated the capacity of Brazilian green propolis extract to act as a gastric protector and anti-ulcer. Animals pre-treated with propolis extract exhibited lower lesion index, ulcer number, and gastric affected area, corroborating the results of Lemos et al. (2007) for *B. dracunculifolia* aerial parts extract. Moreover, green propolis extract decreased the volume of gastric juice, total acidity, and pH. The authors suggest good anti-ulcer activity of green propolis that can be incorporated into ulcer treatment products after pharmacological validation (Barros et al., 2007).

Costa et al. (2018) isolated the major compounds of Brazilian green propolis extract and evaluated their gastroprotective potential in murine gastric ulcers. In the ethanol/HCl-induced ulcer, the compounds artepillin C, baccharin, drupanin (Figure 5), aromadendrin-4′-O-methyl-ether, and kaempferide (Supplementary Figure S3) were orally administered. Artepillin C, drupanin, aromadendrin-4′-O-methyl-ether, and kaempferide decreased the ulcer area, reducing the necrotizing area and the damage to the gastric epithelial architecture. However, baccharin had no significant effect. When animals were treated by intraperitoneal route with a ten times lower dose, all compounds had anti-ulcer results demonstrating a systemic action. After administration of the flavonoids aromadendrin-4′-O-methyl-ether or kaempferide, there was an increase in mucin production resulting in gastroprotection. The compounds did not prevent GSH depletion, but artepillin C and drupanin reduced the lipid hydroperoxides amount at gastric tissue, indicating the prevention of oxidative stress. In addition, all compounds maintained antioxidant enzyme levels similar to those in health mucosa. The indomethacin-induced ulcer model confirmed the gastroprotective and antisecretory activities of the compounds (Costa et al., 2018).

Costa et al. (2020), continuing the research developed by the group (Costa et al., 2018), evaluated the mechanism of propolis extract related to gastric ulcer prevention and healing, and the curative effect of artepillin C. The results indicated that oral pre-treatment with extract avoided gastric damage, reduced SOD activity by about 11% (100 mg/kg) and 26% (300 mg/kg), and increased GST and CAT activity by about 20% and 80%, respectively. Furthermore, extract (300 mg/kg) reduced reactive oxygen species generation and lipid peroxidation in gastric tissue. The daily treatment with extract (300 mg/kg) in the chronic ulcer model promoted a faster healing process and normalized SOD and CAT activities but increased GST activity. The administration of the extract in the two models tested increased the PAS (Periodic Acid of Schiff’s method) staining of mucin, reduced myeloperoxidase activity at the ulcer site, and improved the immunostaining of PCNA (proliferating cell nuclear antigen). However, it did not alter collagen concentration in ulcerated tissue by acetic acid. The extract also showed a natural ability to scavenge DPPH radicals (IC_50_ = 0.56). Extract chemical characterization revealed artepillin C as the main compound (Figure 5) and the administration of this molecule (18 mg/kg) stimulated faster healing of gastric ulcer. The results indicate gastroprotective and gastric healing properties of extract (Costa et al., 2020). Therefore, these findings may contribute to validating their use as preventive and therapeutic approaches.

### 4.3 Antimicrobial

#### 4.3.1 Essential oil

Salazar et al. (2018) studied the essential oil of fresh and dried plant material of *B. dracunculifolia* with the major compounds germacrene D (E)-nerolidol, spathulenol, β-pinene and bicyclogermacrene (Figure 4). The lowest MICs were observed against *S. aureus* strains, 102 μg/ml for ATCC 2593 and 512 μg/ml for a multidrug-resistant clinical isolate. For multidrug-resistant *P. aeruginosa*, the MIC was 813 μg/ml (Salazar et al., 2018). Antimicrobial activity is probably related to the presence of spathulenol (Figure 4) (Tan et al., 2016).

Cazella et al. (2019) evaluated the essential oil of dry aerial parts (leaves and flowers) against microorganisms of interest in food. The main compounds were spathulenol and trans-nerolidol (Figure 4). *S. aureus*, *B. cereus,* and *P. aeruginosa* were the bacteria most susceptible to the essential oil with MIC of 0.5, 1.1, and 1.05 mg/ml, respectively, and MBC of 2.1, 1, 5, and 2.1 mg/ml, respectively, showing weak activity against Enterobacteriaceae (*E. coli* and *Salmonella*). Regarding filamentous fungi, including species of the genus *Penicillium*, *Aspergillus,* and *Trichoderma*, the MIC ranged from 8.43 to 16.87 mg/ml (Cazella et al., 2019).

The essential oil of dried leaves with major compounds such as β-pinene, ledol, spathulenol, and limonene (Figure 4) showed antifungal activity in the control of postharvest fungal rot in grapes caused by the fungi *Botrytis cinerea* and *Colletotrichum acutatum* (Pedrotti et al. al., 2019). Debona et al. (2021) also evaluated the antifungal activity of the essential oil of the leaves of *B. dracunculifolia* against *Alternaria alternata*, a fungus capable of causing damage to dragon fruit. At concentrations above 600 μg/ml, the oil could inhibit fungal growth *in vitro*, a promising option in managing rot caused by this fungus in dragon fruit (Debona et al., 2021).

Monteiro et al. (2022) investigated the antimicrobial activity of essential oil from dry leaves of *B. dracunculifolia*, collected at three different localities in Brazil. The three essential oils showed antimicrobial action for the bacteria studied. Oil from the Midwest region of Brazil was more effective against the bacterial strains tested. For this sample, the MIC was 0.03 mg/ml for *B. cereus*, 0.05 mg/ml for *S. aureus*, *Streptococcus mutans*, *Enterococcus faecalis*, *P. aeruginosa*, *Klebsiella pneumoniae,* and *Salmonella enterica*, and 0.08 mg/ml for *E. coli*. The MBC ranged from 0.05 to 0.1 mg/ml. The major compounds in all samples were sesquiterpenes (59.88–89.29%) and monoterpenes (7.66–38.41%). In the Central-West region, the majority were (E)-nerolidol (28.15%) and spathulenol (17.68%) (Figure 4). In the Southeast region, they were limonene (19.36%) (E)-nerolidol (12.75%) and bicyclogermacrene (10.76%) (Figure 4) and in the southern region bicyclogermacrene (14.21%) (E)-nerolidol (13.95%) and limonene (10.49%) (Figure 4). Bicyclogermacrene, after enzymatic oxidation, gives rise to spathulenol (Monteiro et al., 2022).

The commercial essential oil of *B. dracunculifolia* from the company *Harmonia* Natural (Canelinha, SC, Brazil), having as main components nerolidol, beta-pinene and d-limonene (Figure 4) (Timbé et al., 2021), has antimicrobial activity against the Gram-positive bacteria studied, the largest inhibition halo was against *Bacillus subtilis* (23.6 mm). Not showing activity against Gram-negative bacteria and yeasts (Timbé et al., 2021). This same research group also evaluated the antimicrobial activity of nanoparticles containing *B. dracunculifolia* oil (*Harmonia* Natural - Canelinha, SC, Brazil) (Timbé et al., 2020). First, the MBC of the oil was analyzed against *Listeria monocytogenes* (0.56 mg/ml), *S. aureus* (0.28 mg/ml), *B. cereus* (0.14 mg/ml) and *S. Enteritidis* (1.13 mg/ml). Afterward, the contact test of the oil with the bacteria studied was carried out in a liquid culture medium. After 4 h of contact of the essential oil with *S. aureus*, *B. cereus,* and *L. monocytogenes,* and *S.* Enteritidis, there were no more viable cells. The reduction achieved by the nano encapsulated oil was 2 h of incubation for *B. cereus*, 10 h for *L. monocytogenes,* and 24 h for *S. aureus* and *S.* Enteritidis, suggesting a gradual release of compounds with antimicrobial activity (Timbé et al., 2020).

#### 4.3.2 Extract

Bonin et al. (2020) studied the antimicrobial activity of the hydroalcoholic extract (70%) (dry plant material - plant parts not reported) of *B. dracunculifolia* against bacteria. The minimum inhibitory concentrations (MIC) and bactericidal concentrations (MBC) found, respectively, were 125 μg/ml and 250 μg/ml for *S. aureus* and *B. subtilis,* 250 μg/ml and 500 μg/ml for *B. cereus.* For the *Salmonella enterica* serovar Enteritidis, the MIC and MBC values were 1,000 μg/ml. The antimicrobial activity against Gram-positive bacteria may be associated with the compound naringenin (Supplementary Figure S3) present in the extract (Bonin et al., 2020), a compound found by Zuccolotto et al. (2016) when they analyzed the antimicrobial action of other plants belonging to the genus *Baccharis* against Gram-positive bacteria.

Casagrande et al. (2018) found equal antimicrobial activity of the hydroalcoholic extract (40%) and acetone extract (40%), from the aerial part of the plant, against the Gram-positive bacteria studied. For *S. aureus* MIC of 12.75 mg/ml and for *B. cereus* MIC of 3.19 mg/ml. For Gram-negative bacteria, the activity of the extracts was lower. Still, attention is drawn to the antimicrobial activity of the hydroalcoholic extract against *Pseudomonas aeruginosa*, with MIC and MBC of 51 mg/ml and resistance to ampicillin (Casagrande et al., 2018). This same research group evaluated the addition of the hydroalcoholic extract at different concentrations in a film based on poly (vinyl alcohol) and starch (Casagrande et al., 2021). For this, they lyophilized the extract and analyzed its antimicrobial and antioxidant characteristics before producing the film. The antimicrobial activity against Gram-positive bacteria, found by Casagrande et al. (2021), was better when compared to the liquid extract (Casagrande et al., 2018). For *S. aureus* and *B. cereus* the MIC was 1.49 mg/ml, and 0.37 mg/ml, respectively. For *P. aeruginosa*, the MIC was higher than the concentrations analyzed. After being added to the film, the antimicrobial activity was reduced and even inhibited for the microorganisms mentioned above (Casagrande et al., 2021).

Hydroalcoholic extract (70%) of dried leaves showed antimicrobial activity against *Staphylococcus pseudintermedius*, with MIC of 0.312 mg/ml and MBC of 2.5 mg/ml, in addition to having the ability to inhibit biofilm formation *in vitro* (Barbosa et al., 2022). This activity may be related to phenolic acid derivatives and flavonoids (Barbosa et al., 2022). The commercial hydroalcoholic extract of *B. dracunculifolia* was obtained from Ciclo Farma Indústria Química Ltda (Serrana, SP, Brazil), with the majority of artepillin C (Figure 5), rutin, and caffeic acid (Supplementary Figure S4) has been evaluated by Timbé et al. (2021), presenting antimicrobial activity against all tested bacteria, with the highest inhibition halo for *B*. *cereus* (20.5 mm). The material evaluated did not show activity against Gram-negative bacteria and yeasts (Timbé et al., 2021). Veiga et al. (2017) analyzed ethanolic and hexane extracts from leaf shoots. The ethanol extract presented MIC_90_ between 256.7 and 770.1 μg/ml for the *S. aureus* strains studied, while the hexane MIC_90_ was between 197 and 394 μg/ml. Even the ethanol extract having a higher content of phenols and flavonoids had a lower antimicrobial capacity (Veiga et al., 2017).

Bernardes et al. (2022) tested the antimicrobial activity of hydroalcoholic extracts from aerial parts and roots and trichome washing of *B. dracunculifolia* against bacterial and fungal strains. The best results were against *S. aureus* (MIC 200 μg/ml for the extract of aerial parts and trichome wash) and *Trichophyton mentagrophytes* (MIC 200 μg/ml for the extract of aerial parts). In addition, when the substances isolated from the extracts were evaluated, baccharin (Figure 6) showed activity against *S. aureus* and *T. mentagrophytes*, and isosakuranetin (Supplementary Figure S3) against *Salmonella choleraesuis* and *T. mentagriphytes*. According to the results found in this work, *B. dracunculifolia* is a potential alternative as a sanitizer against pathogenic fungi and bacteria (Bernardes et al., 2022).

Assumpção et al. (2022) evaluated the antimicrobial activity of the hydroalcoholic extract of aerial parts of *B. dracunculifolia* against strains of *S. aureus* isolated from mastitis. The MIC for two of the clinical strains was 1.25 mg/mL. As for biofilm formation, the extract showed better results for consolidated biofilms than new biofilms (Assumpção et al., 2022). The ethanolic extract (99.8%) produced from leaves, with the presence of cardiotonic heterosides, steroids, phenolic compounds, flavonoids, tannins, coumarins, and triterpenes, at a concentration of 2000 µL/100 ml, inhibited by 13.5% the growth of *Rhizoctonia solani* (Dilkin et al., 2022).

#### 4.3.3 Green propolis

Barbosa et al. (2022) evaluated the hydroalcoholic extract (70%) of green propolis. They found antimicrobial activity against *Staphylococcus pseudintermedius*, with MIC of 0.156 mg/ml and MBC of 0.312 mg/ml, with a chemical composition similar to the extract of the leaves of *B. dracunculifolia* (derived from phenolic acids and flavonoids) (Barbosa et al., 2022).

Ethanolic and hexane extracts of green propolis had their antimicrobial activity studied (Veiga et al., 2017). Phenols and flavonoids were found only in the ethanol extract, in addition to the higher amount of artepillin C (Supplementary Figure S6A). Even with this difference in composition, the hexane extract showed better antimicrobial activity against the *S. aureus* strains analyzed (MIC_90_ between 78.4 and 392.0 μg/ml) (Veiga et al., 2017). Assumpção et al. (2022) studied the biofilm formation and antimicrobial action of the hydroalcoholic extract of green propolis against mastitis isolates. Propolis presented a similar result to the extract of aerial parts. The MIC for the clinical strains was between 1.25 and 2.5 mg/ml, showing better results against consolidated biofilms (Assumpção et al., 2022).

**FIGURE 11:**
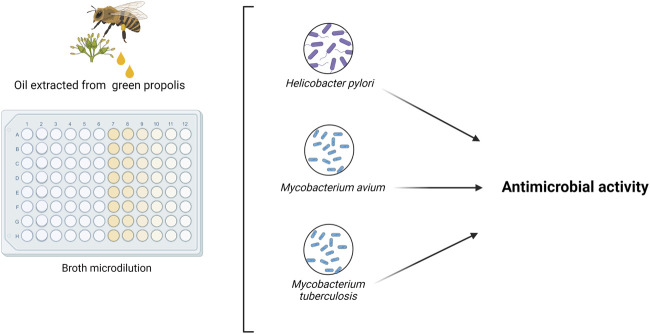
Antimicrobial activity of the oil extracted from *B. dracunculifolia* green propolis (Quintino et al., 2020).

Bittencourt et al. (2015) correlated the free radical scavenging activity (IC50) and MIC to the presence of total phenolic compounds in Brazilian green propolis. They found that the IC50 and MIC values were associated with antioxidant and antibacterial activities (Bittencourt et al., 2015). Machado et al. (2016) tested ethanolic extract of green propolis against *S. aureus* and *Escherichia coli*. They identified a negative correlation between phenols concentration in the extracts and MIC. Jug et al. (2014) also evaluated the antibacterial and antifungal efficiency of green propolis extracts obtained by different extraction techniques and demonstrated that the ethanolic extract showed the best antimicrobial action. Quintino et al. (2020) verified the antimicrobial activity of the oil extracted from fresh green propolis (Figure 11), with carvacrol being the compound with the highest concentration (20.7%), finding a MIC of 6.25 μg/ml against *Helicobacter pylori*, 62.5 μg/ml against *Mycobacterium avium* and 64 μg/ml for *Mycobacterium tuberculosis* (Quintino et al., 2020).

### 4.4 Antioxidant

#### 4.4.1 Essential oil

Tomazzoli et al. (2021) analyzed the antioxidant activity of the essential oil of fresh leaves of *B. dracunculifolia* collected in ten locations in Paraná - Brazil. The method used was DPPH, with activity varying between 27.78% and 91.67%. The oils were predominantly composed of oxygenated sesquiterpenes, monoterpene hydrocarbons, and sesquiterpene hydrocarbons. With the majority in all ten populations, β-pinene and (E)-nerolidol (Figure 4) (Tomazzoli et al., 2021), compounds that have already been reported with antioxidant activity (Yu et al., 2017; Chen et al., 2018). Monteiro et al. (2022), analyzing oil from leaves of plants collected from three regions of Brazil, found that oil from the Center-West region of Brazil showed larger antioxidant activity by the DPPH methods (50.82% inhibition) and ABTS (113.63 mM TE/ml) concerning oils from the Southeast and South regions. Oils from the Center-West and South regions of Brazil had higher levels of phenolic compounds compared to the oil from the Southeast region of Brazil. One of the major compounds common to the three samples was (E)-nerolidol (Figure 4) (Monteiro et al., 2022).

Paroul et al. (2016) evaluated the antioxidant activity, by the DPPH method, of essential oil and aqueous extract of plant material of *B. dracunculifolia*. The antioxidant activity of the aqueous extract was 100 times greater than that obtained with the essential oil, which is possibly related to the greater antioxidant action of the compounds present in this type of extract, probably because they are water-soluble compounds (data not provided) (Paroul et al., 2016).

#### 4.4.2 Extract

Casagrande et al. (2018), evaluating hydroalcoholic extract and acetone extract, verified antioxidant activity by the methods of DPPH (2,2-diphenyl-1-picrylhydrazyl), ABTS (2.2′-azinobis-(3-ethylbenzothiazoline-6-sulfonic acid), FRAP (ferric reducing/antioxidant power) which may be associated to the phenolic compounds in its composition, with ferulic acid (hydroxycinnamic acid) (Supplementary Figure S4), being the main component (Casagrande et al., 2018). Casagrande et al. (2021) evaluated the same lyophilized hydroalcoholic extract as a pre-test for addition to a film based on poly (vinyl alcohol) and starch. The antioxidant activity of the DPPH, ABTS, and FRAP methods was better at 389%, 322%, and 533%, respectively, when compared to the liquid extract (Casagrande et al., 2018). The embedded film maintained the antioxidant action when analyzed by the DPPH method (Casagrande et al., 2021).

Guimarães et al. (2012) evaluated the antioxidant activity *in vitro* and hepatic mitochondria, isolated from rats, of the glycolic extract from the leaves of *B. dracunculifolia*. The concentrations of total phenols and flavonoids were 21.18 µM (gallic acid equivalents) and 13.64 µM (quercetin equivalents), respectively. Caffeic acid, *p*-coumaric acid, cinnamic acid (Supplementary Figure S4), aromadendrin, isosakuranetin (Supplementary Figure S3), and artepellin C (Figure 5) were the compounds identified. By the DPPH method, the EC_50_ was 0.005%, by superoxide elimination, the EC_50_ was 0.0732%, in addition, the extract was able to chelate 77.57% of Fe^2+^. In mitochondria, there was a decrease in the basal generation of H_2_O_2_ and ROS production induced by Fe^2+^- or t-BuOOH. There was also prevention of lipid oxidation of mitochondrial membranes, thiol protein groups (Guimarães et al., 2012).

**FIGURE 12:**
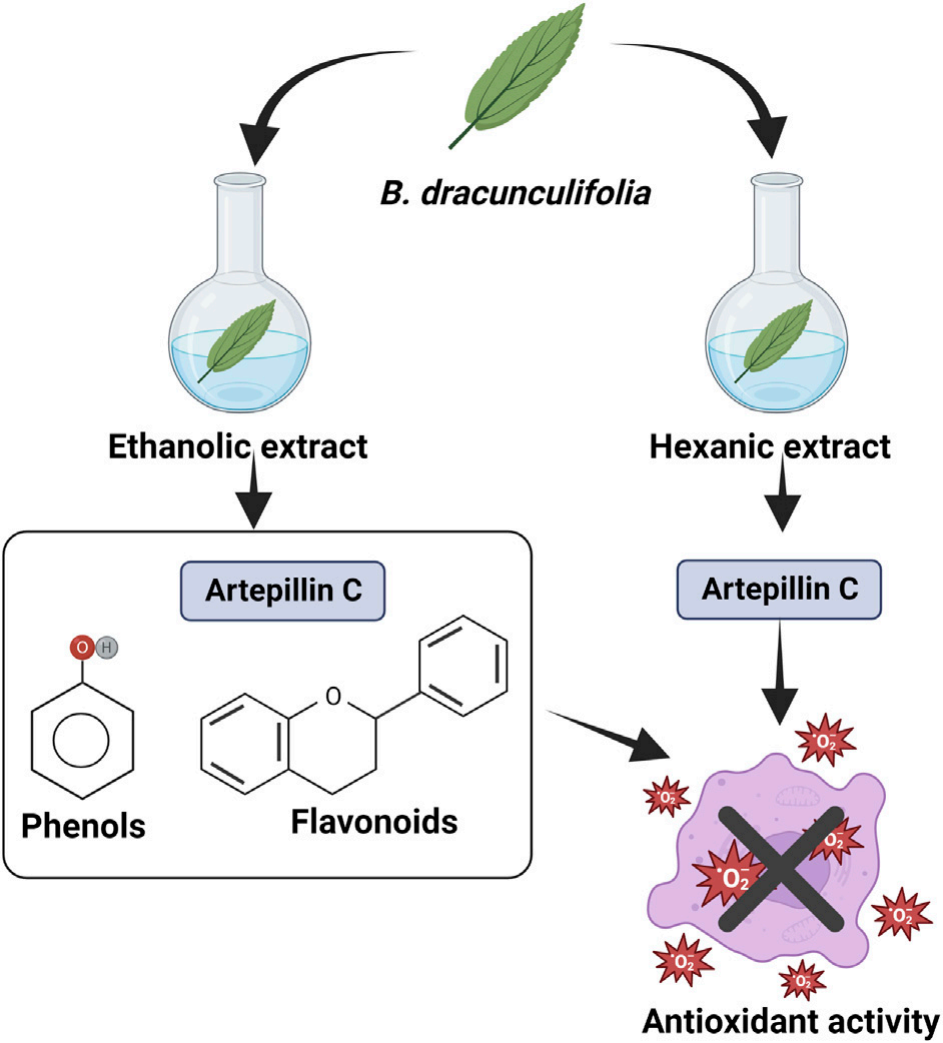
Antioxidant activity of ethanolic and hexane extracts from *B. dracunculifolia* leaves (Veiga et al., 2017).

The antioxidant activity of ethanolic and hexane extracts from *B. dracunculifolia* sprouts (Figure 12), and green propolis was evaluated by the DPPH method. The IC_50_ values were 13.09 μg/ml for ethanolic extract of green propolis, 95.86 μg/ml for hexane extract of green propolis, 124.49 μg/ml for ethanolic extract of *B. dracunculifolia* and 141.45 µg/ml for *B. dracunculifolia* hexane extract. It is observed that among all samples, the ethanolic extract has the larger activity. This extract has phenols and flavonoids in its composition (Veiga et al., 2017).

#### 4.4.3 Green propolis

The antioxidant activity of green propolis was reported by Osés et al. (2016), Cao et al. (2017), and Braakhuis (2019). According to the authors, polyphenols, one of the main propolis compounds, have a chemical structure with potential scavenging free radicals. Furthermore, the flavonoids in propolis are potent antioxidants, capable of scavenging free radicals and thus protecting cell membranes against lipid peroxidation (Osés et al., 2016; Cao et al., 2017; Braakhuis, 2019).

The antioxidant activity of the oil extracted from fresh green propolis was evaluated by the DPPH and ABTS methods, obtaining IC_50_ of 23.48 μg/ml and 32.18 μg/ml, respectively. In this study, the major compounds were carvacrol (20.7%), acetophenone (13.5%), spathulenol (11.0%) (E)-nerolidol (9.7%) and β-caryophyllene (6.2%) (Figure 4) (Quintino et al., 2020).

With the evidence of the antioxidant activity of *B. dracunculifolia*, Zanela et al. (2021) produced a biodegradable film for food based on PBAT—poly (butylene adipate co-terephthalate) and cassava starch using glycerol as a plasticizer. *B. dracunculifolia* leaf powder was added to the film, and the antioxidant, physical and chemical properties were found to be ideal for use as biodegradable food packaging (Zanela et al., 2021).

### 4.5 Antiviral

The inhibitory effects of plant extracts on the replication of various viruses have been reported in several studies. Viruses such as herpes simplex (HSV), human immunodeficiency (HIV), hepatitis B (HBV) and severe acute respiratory syndrome (SARS) were strongly inhibited by various plant extracts.

Búfalo et al. (2009) evaluated the antiviral activity of the extract, essential oil, green propolis, and isolated compounds caffeic and cinnamic acids (Supplementary Figure S4) from *B. dracunculifolia* against poliovirus type 1 (PV1) replication in HEp-2 cells (human laryngeal epidermoid carcinoma cells). The results indicated that the greatest inhibition of virus replication was achieved by the extract of *B. dracunculifolia* with 74.0%, followed by green propolis (52.2%), essential oil (33.3%), cinnamic acid (29, 8%) and caffeic acid (26.7%). The most efficient concentrations of *B. dracunculifolia* extract and caffeic and cinnamic acid compounds were 25 µg/100 μL, followed by green propolis (10 µg/100 µL) (Búfalo et al., 2009).

The antiviral activity of ethanol extract of green propolis obtained from *B. dracunculifolia* was tested by Shimizu et al. (2008) on the propagation of influenza A/PR/8/34 (H1N1) virus in Madin-Darby cells (MDCK) obtained from the kidney of dogs and female DBA/2 Cr mice. The anti-influenza efficacy of the extract at a concentration of 10 mg/kg was confirmed in a dose-dependent manner in mice. At this concentration, there was a significant reduction in the virus yield in bronchoalveolar lavage fluids from the lungs of infected mice when compared to the control group. It was observed that the reduction in virus yields by the extract at a concentration of 10 mg/kg corresponded significantly to those induced by oseltamivir at a concentration of 1 mg/kg at four post infection (Shimizu et al., 2008). In this same line of research, Shimizu et al. (2011) administered ethanolic extracts (10 mg/kg) of *B. dracunculifolia* orally in mice infected with Herpes virus type 1 (HSV-1). The extract significantly reduced the titers of the virus in the brain and/or in the skin from the fourth day of use, without showing toxicity. A significant increase in the production of interferon-gamma (IFN-γ) by the HSV-1 antigen was also observed in splenocytes from mice infected with HSV-1 (Shimizu et al., 2011).

### 4.6 Other activities

Luchesi et al. (2022) investigated the antiphytopathogenic activity of essential oil extracted from fresh leaves of *B. dracunculifolia* against the fungus *Fusarium graminearum*. The oil was tested at concentrations of 8.0 and 4.0 μL/ml, inhibiting fungal growth by 57.1% and 49.5%, respectively, after 96 h. Inhibition of the fungus may be associated with the presence of cis- and trans-nerolidol, c-elemene, d-limonene and spathulenol (Figure 4), considered by the authors as the major compounds in the essential oil (Luchesi et al., 2022).

Cazella et al. (2020) analyzed the acaricidal activity of the essential oil extracted from the leaves and flowers of *B. dracunculifolia*, finding the sesquiterpenes nerolidol and spathulenol as the main compounds (Figure 4). The oils (leaves and flowers) were tested at stages comprising the parasitic and free-living life cycle of the bovine tick *Rhipicephalus microplus* (Acari: Ixodidae). Both essential oils at a concentration of 500 mg/ml effectively controlled the hatchability of eggs, reducing the oviposition capacity and the number of adult and larval ticks. The lethal concentration (CL_99,9_) of essential oils (leaves and flowers) on the larvae was 35–37 mg/ml, respectively. The authors also investigated the activity of the oils in the tick free life cycle (*ex situ* test), indicating that at concentrations of 11–14 mg/ml there were 85 and 95% efficacy, respectively (Cazella et al., 2020).

Lage et al. (2015) also evaluated the chemical composition and acaricidal activity of the essential oil extracted from the aerial parts of *B. dracunculifolia* and the pure compounds nerolidol and (R)-(+)-limonene on the larvae and engorged females of *Rhipicephalus microplus*. For larvae, the “Larval packet test” was performed and for engorged females, the “adult immersion test”. The most efficient result was the action of nerolidol (Figure 4) on tick larvae, causing more than 90% mortality at a concentration of 10.0 mg/ml. Essential oil and nerolidol also acted in the immersion test of adults, causing a reduction in the quantity and quality of eggs produced, with inhibition of hatchability of 96.3% and 90.3% at concentrations of 60.0 and 50.0 mg/ml, respectively (Lage et al., 2015).

As an insecticide, the essential oil of this plant has already been tested against third-stage larvae of *Cochliomyia macellaria* by Chaaban et al. (2018), where this demonstrated as an ecological alternative against this fly because, in addition to being highly larvicidal (2.47 μL/cm^2^), the hatched larvae generated adults with deformities. Furthermore, from the essential oil of the leaves of *B. dracunculifolia*, Alves et al. (2018) verified larvicidal action on *Culex quinquefasciatus*. The essential oil (EO) was tested at concentrations of 25, 50, 100 and 200 mg/L, with a lethal concentration (LC_50_) of 34.45 mg/L for the EO. Histological changes were observed in the midgut, reduction of glucose and acetylcholinesterase levels in larvae exposed to EO, indicating increased levels of triacylglycerides and total proteins, confirming that the EO of *B. dracunculifolia* causes destabilization in the larva, leading to histological changes, dysregulation metabolism and, consequently, its death. The authors suggest that this effect is due to the presence of sesquiterpenes such as spathulenol and nerolidol (Figure 4), which are used to control insects and as an acaricide (Cazella et al., 2020).

Seugling et al. (2019) developed nanoemulsions containing essential oil of *B. dracunculifolia* in five different concentrations (5.0; 7.5; 10.0; 13.5 and 15.0%) (w/v). The tests consisted of using the nanoemulsions immediately after preparation and after 120 days of storage on *Cochliomyia hominivorax* larvae. The authors verified that the formulations used immediately after preparation, provided mortality of 28, 48, 70, 84 and 97%, respectively, while for the stored formulations the mortality was 17; 36; 51; 81 and 92%, respectively. The authors found no significant difference in the action of nanoemulsions in terms of storage time, suggesting that nanoemulsions of essential oil of *B. dracunculifolia* can be considered a promising alternative for the treatment and control of myiasis. caused by *C. hominivorax* (Seugling et al., 2019).

#### 4.6.1 Green propolis

Green propolis is known for its biological properties, and in this sense, Dantas Silva et al. (2017) investigated the activity of ethanolic extracts (80%) of green propolis from different regions of Brazil on epimastigotes of *Trypanosoma cruzi* “Y strain”. The authors verified an inhibitory effect of the extracts on the growth of cultures at concentrations of 75 and 300 mg ml-1 on the growth of epimastigotes of the Y strain of *T. cruzi*. This inhibition was observed in the first 24 h of the experiment, indicating green propolis as an alternative therapeutic treatment against Chagas disease, a deadly disease considered neglected, with few therapeutic resources (Dantas Silva et al., 2017).

There is considerable evidence of anticancer properties of brazilian green propolis (Ahn et al., 2007; Szliszka et al., 2011). The use of propolis has been recommended as a complementary therapy for the treatment of various types of cancer, including bladder, blood, brain, breast, colon, head and neck, kidney, liver, pancreas, prostate and skin (Patel, 2016). Compounds isolated from propolis are patented drugs for the treatment of cancer (Chuang et al., 2016), and in this sense, Matsuno (1995) isolated a new clerodane-type diterpene (Supplementary Figure S2). These authors verified in their research that this compound inhibited the growth of hepatoma cells and stopped tumor cells in the S phase, which corresponds to the period of DNA synthesis (Matsuno, 1995).

Anticancer activities of ethanolic extracts of green propolis were also evaluated by Machado et al. (2016). Propolis extracts were obtained by supercritical (SCO2) and ethanolic (EtOH) extraction, whose samples were collected in different regions of Brazil. Assays were performed on B16F10 tumor cells, evaluating the antiproliferative effect, using two concentrations of extract (50 and 100 μg/ml). Cell proliferation was measured after 24 and 48 h, showing a significant inhibition of cell proliferation at the two evaluated times. We highlight the results obtained for the sample of green propolis collected in the state of Paraná-Brazil. This extract recorded the highest concentration of artepillin C (Supplementary Figure S6A) and p-coumaric acid (Supplementary Figure S4). Other important results in relation to artepillin C were found by Kimoto et al. (2001), who demonstrated its antileukemic effect.

Studies indicate that propolis has a complex chemical composition. For this reason, isolated compounds should be investigated in vitro and *in vivo* tumor assays, as well as the synergistic effects between them (Moise and Bobis, 2020). It is believed that propolis can exert a direct effect on different tumor cells in vitro assays, and its administration in animals or humans will depend on factors such as solubility and systemic bioavailability for this action to be achieved. Thus, the antitumor activity of propolis may occur mainly due to its immunomodulatory action, exerting chemopreventive or therapeutic effects (Watanabe et al., 2011).

The inhibitory effect of caffeic acid phenethyl ester, an active component of propolis, was demonstrated by Liao et al. (2003) on angiogenesis, tumor invasion, and metastatic lung capacity of colon carcinoma cell (CT26). According to the authors, the compound caffeic acid phenethyl ester prolonged the survival of mice implanted with CT26 cells, demonstrating its potential as an antimetastatic agent. Furthermore, Lee et al. (2003) reported that concentrations between 10–400 μM of CAPE provided a dose-dependent effect on the cytotoxicity of C6 glioma cells, promoting a reduction in viability to 42% when compared to the control. Lee et al. (2003) reported that concentrations between 10–400 μM of CAPE provided a dose-dependent effect on the cytotoxicity of C6 glioma cells, promoting a reduction in viability to 42% when compared to the control. Lee et al. (2005) investigated the effect of CAPE on oral cancer using a cultured cancer cell line (squamous cell carcinoma, SAS; oral squamous cell carcinoma-Meng 1, OEC-M1) and normal human oral fibroblasts (NHOF). The results demonstrated cytotoxic effects on tumor cells, demonstrating the arrest in replication of OEC-M1 cells in the G2/M phase that occurs after DNA duplication and before cell division. Based on the results, the authors suggest that these compounds may be useful in oral cancer chemotherapy (Lee et al., 2005).

Information on the pharmacology and bioactive compounds of *B. dracunculifolia* and green propolis are summarized in Table 3.

**TABLE 3:** Summary of biological activities and bioactive compounds reported in various studies, including plant parts and extracting solvent.

Sample	Plant part	Extracting solvent	Bioactive compounds	Biological activities	References
essential oil	leaves	-	sesquiterpenes	anti-inflammatory	Brandenburg et al. (2020)
extract	aerial parts	ethyl acetate	caffeic acid, *p*-coumaric acid, drupanin, baccharin, artepillin C	anti-inflammatory	Cestari et al. (2011)
extract	leaves	Ethanol	caffeic acid	anti-inflammatory	Figueiredo-Rinhel et al. (2019)
extract	aerial parts	Ethanol 99.5%	-	anti-inflammatory	Lima et al. (2020)
extract	leaves	Ethanol 70%	-	anti-inflammatory	França et al. (2022)
green propolis^©^ extract	-	Ethanol 70%	caffeic acid, *p*-coumaric acid, *trans*-cinnamic acid, aromadendrin, artepillin C	anti-inflammatory	Hori et al. (2013)
green propolis^©^ extract	-	Ethanol 70%	caffeic acid, *p*-coumaric acid, aromadendrin, drupanin, artepillin C	anti-inflammatory	Mariano et al. (2018)
green propolis extract	resin	Ethanol 95%	*p*-coumaric acid, quercetin, kaempferol, galanin, artepillin C	anti-inflammatory	Wang et al. (2018)
green propolis^©^ extract	-	hydroalcoholic	*p*-coumaric acid, rutin, pinobanksin, quercetin, kaempferol, apigenin, pinocembrin, pinobanksin-3-acetate, chrysin, galangin, techtochrysin, artepillin C, baccharin	anti-inflammatory	Miranda et al. (2019)
green propolis^©^ extract	-	Ethanol 70%	4′-methoxy pinobanksin, 3-isoprenyl-*p*-coumaric acid, 3-hydroxy-2,2-dimethy-8-prenylchromane-6-propenoic, pinocembrin, kaempferide, quercetin-dimethyl ether, diisoprenyl-*p*-coumaric acid isomer, 3-prenyl-4-(dihydrocinnamoyloxy)-cinnamic acid, E)-3-[2,3-dihydro-2-(1-methylethenyl)-7-prenyl-5-benzofuranyl]-2-propenoic acid, triterpenes, artepillin C	anti-inflammatory	Xu et al. (2020)
green propolis^©^ extract	-	Ethanol 95%	chlorogenic acid, caffeic acid, isochlorogenic acid A, isochlorogenic acid C, myricetin, quercetin, kaempferol, apigenin, pinocembrin, caffeic acid phenethyl ester, galangin, artepillin C	anti-inflammatory	Yuan et al. (2020)
green propolis extract	resin	Ethanol 90%	*p*-coumaric acid, baccharin	anti-inflammatory	Ferreira et al. (2021)
essential oil	leaves	-	mono- and sesquiterpenes: nerolidol	antiulcerogenic	Klopell et al. (2007)
essential oil	leaves	-	α-pinene, β-pinene, limonene, *trans*-caryophillene, aromadendrene, α-humulene, germacrene-D, bicyclogermacrene, δ-cadinene, nerolidol, spathulenol, viridiflorol, α-muurolol	antiulcerogenic	Massignani et al. (2009)
extract	aerial parts	Ethanol 70%	caffeic acid, *p*-coumaric acid, ferulic acid, aromadendrin-4′O-methyl ether, isosakuranetin, artepelin C, baccharin	antiulcerogenic	Lemos et al. (2007)
extract	aerial parts	Ethanol 70%	ferulic acid, *p*-coumaric acid, caffeic acid, artepillin C, baccharin, aromadendrin-4′-O-methyl ether	antiulcerogenic	Costa et al. (2019)
extract	aerial parts	Ethanol 70%	*p*-coumaric acid	antiulcerogenic	Boeing et al. (2021)
green propolis extract	resin	Ethanol 70%	caffeic acid, *p*-coumaric acid, drupanin, isosakuranetin, aromadendrin, artepillin C	antiulcerogenic	Barros et al. (2007)
green propolis^©^ extract	resin	Ethanol 70%	aromadendrin-4′-O-methyl-ether, kaempferide, drupanin, baccharin, artepillin C	antiulcerogenic	Costa et al. (2018)
green propolis^©^ extract	resin	Ethanol 70%	caffeic acid, *p*-coumaric acid, ferulic acid, cinnamic acid, aromadendrin-4′-O-methyl ether, isosakuranetin, drupanin, artepillin C, baccharin	antiulcerogenic	Costa et al. (2020)
extract	-	Ethanol 70%	germacrene B, naringenin, Kaempferol, artepillin C, α-pinene, hydroxycinnamic acid, apigenin, kaempferide, limonene, phenylethanol, β-caryophyllene	antimicrobial	Bonin et al. (2020)
extract	-	Ethanol 40% and acetone 40%	ferulic acid (hydroxycinnamic acid), catechin, epigallocatechin, quercetin, rutin	Antimicrobial and antioxidant	Casagrande et al. (2018)
lyophilized extract + poly (vinyl alcohol) and starch	aerial parts	Ethanol 40%	catechin, ferulic acid, apigenin, kaempferol, epigallocatechin, myricetin	Antimicrobial and antioxidant	Casagrande et al. (2021)
extract	leaves	Ethanol 70%	caffeic acid, coumaric acid, cinnamic acid, dihydrokaempferide drupanin, artepillin C, baccharin, 2,2-dimethyl-6-carboxyethyl-2H-1-benzopyran	antimicrobial	Barbosa et al. (2022)
extract^©^	-	hydroalcoholic	artepillin C, rutin, caffeic acid	antimicrobial	Timbé et al. (2021)
extract	leaf shoots	Ethanol 95%	phenols, flavonoids, artepillin C	antimicrobial and antioxidant	Veiga et al. (2017)
extract	leaf shoots	hexane	artepillin C	antimicrobial and antioxidant	Veiga et al. (2017)
extract	aerial parts	Ethanol 96%	caffeic acid, coumaric acid, ferulic acid, baccharin, drupanin, artepillin C, hispidulin, aromadendrin-4′-O-methyl-ether	antimicrobial	Bernardes et al. (2022)
extract	roots and trichome washing	Ethanol 96%	isosakuranetin, 3-O–methyl–kaempferol, baccharis oxide friedelanol	antimicrobial	Bernardes et al. (2022)
extract	aerial parts	Ethanol 70%	-	antimicrobial	Assumpção et al. (2022)
extract	leaves	Ethanol 99.8%	cardiotonic heterosides, steroids, phenolic compounds, flavonoids, tannins, coumarins, triterpenes	antimicrobial	Dilkin et al. (2022)
essential oil	-	-	germacrene D, (E)-nerolidol, spathulenol, β-pinene, bicyclogermacrene	antimicrobial	Salazar et al. (2018)
essential oil	leaves and flowers		spathulenol, trans-nerolidol, heptacosane, β-pinene, bicyclogermacrene, trans-caryophyllene, germacrene D, a-muurolol	antimicrobial	Cazella et al. (2019)
essential oil	leaves	-	β-pinene, ledol, spathulenol, limonene	antimicrobial	Pedrotti et al. (2019)
essential oil	leaves	-	-	antimicrobial	Debona et al. (2021)
essential oil	leaves	-	(E)-nerolidol, spathulenol, limonene, bicyclogermacrene	antimicrobial and antioxidant	Monteiro et al. (2022)
essential oil^©^	-	-	nerolidol, β-pinene, d-limonene, caryophyllene, elixene, a-pinene, germacrene D and spatulenol	antimicrobial	Timbé et al. (2021)
nanoparticles containing essential oil	-	-	nerolidol, beta-pinene, d-limonene	antimicrobial	Timbé et al. (2020)
green propolis extract	resin	Ethanol 70%	caffeic acid, coumaric acid, cinnamic acid, dihydrokaempferide drupanin, artepillin C, baccharin, 2,2-dimethyl-6-carboxyethyl-2H-1-benzopyran	antimicrobial	Barbosa et al. (2022)
green propolis extract	resin	Ethanol 95%	phenols, flavonoids, artepillin C	antimicrobial and antioxidant	Veiga et al. (2017)
green propolis extract	resin	hexane	artepillin C	antimicrobial and antioxidant	Veiga et al. (2017)
green propolis extract	resin	Ethanol 70%	-	antimicrobial	Assumpção et al. (2022)
green propolis extract	resin	Ethanol 95%	phenolic compounds, nerolidol, δ-cadinene, γ-muurolene, and caryophyllene, caryophyllene oxide	antioxidant and antibacterial	Bittencourt et al. (2015)
green propolis^©^ extract	resin	supercritical, ethanolic	artepillin C, p-coumaric acid	antimicrobial	Machado et al. (2016)
green propolis extract	resin	Ethanol	-	antimicrobial	Jug et al. (2014)
green propolis extract	resin	-	carvacrol, acetofenona, espatulenol, (E)-nerolidol, β-cariofileno	antimicrobial and antioxidant	Quintino et al. (2020)
extract	leaves	glycolic	caffeic acid, p-coumaric acid, cinnamic acid, aromadendrin, isosakuranetin, artepellin C	antioxidant	Guimarães et al. (2012)
essential oil	leaves	-	β-pinene, (E)-nerolidol	antioxidant	Tomazzoli et al. (2021)
essential oil and extract	-	water	-	antioxidant	Paroul et al. (2016)
extract, essential oil, green propolis	dried leaves and resin	-	-	antiviral	Búfalo et al. (2009)
green propolis extract	resin	Ethanol	-	antiviral	Shimizu et al. (2008)
extract	-	Ethanol	-	antiviral	Shimizu et al. (2011)
essential oil	leaves	-	cis- and trans-nerolidol, c-elemene, d-limonene, spathulenol	antiphytopathogenic	Luchesi et al. (2022)
essential oil	leaves, flowers	essential oil	nerolidol, spathulenol	acaricidal	Cazella et al. (2020)
essential oil	aerial parts	-	-	acaricidal	Lage et al. (2015)
essential oil	-	-	-	insecticide	Chaaban et al. (2018)
essential oil	-		spathulenol, nerolidol	larvicidal	Alves et al. (2018)
nanoemulsions containing essential oil	-	-	-	larvicidal	Seugling et al. (2019)
green propolis extract	resin	Ethanol 80%	-	antiparasitic	Dantas Silva et al. (2017)
green propolis	resin	-	-	anticancer	Ahn et al., 2007, Szliszka et al. (2011)
green propolis	resin	-	-	anticancer	Patel (2016)
green propolis	resin	-	clerodane-type	anticancer	Matsuno (1995)
green propolis^©^ extract	resin	supercritical, ethanolic	artepillin C, *p*-coumaric acid	anticancer	Machado et al. (2016)
green propolis	resin	-	artepillin C	antileukemic	Kimoto et al. (2001)
green propolis	resin	-	caffeic acid phenethyl ester	anticancer	Liao et al. (2003), Lee et al. (2003), Lee et al. (2005)

(-): not reported by authors.

## 5 Toxicology

Rodrigues et al. (2009) evaluated the mutagenic and genotoxic effect of aqueous extract of aerial parts of *B. dracunculifolia* in female mice by gavage at concentrations of 0.5 g/kg, 1.0 g/kg or 2.0 g/kg for 3 days. Lethargy, decreasing locomotor activity, and exploratory behavior were the clinical signs of toxicity observed, but no animal died. In addition, the authors suggested genotoxic effects at the highest dose tested (2.0 g/kg) with a damage frequency (%) of 74.3%, which found a significant increase in DNA damage in the liver tissues and blood of the treated mice. The toxicity was confirmed by a decrease in the polychromatic erythrocytes: normochromic erythrocytes (PCE:NCE) ratio de 1.42 (the control group in saline) to 0.93 in the treated group, indicating some cytotoxicity to bone marrow. In contrast, Bonin et al. (2020) found that the hydroalcoholic extract of *B. dracunculifolia* showed low or null cytotoxicity against Vero cells, whose CC50 (cytotoxic concentration for 50% of the cells) after 48 h was 628.7 ± 279.2 μg/ml (Rodrigues et al., 2009).

## 6 Conclusion

*B. dracunculifolia* is a medicinal ethnobotanical plant native to South America and important in the production of green propolis. Due to its rapid vegetative development, this species is used in the restoration of degraded areas, is known as a “benefactor” plant facilitating the development of other plant species, being used in reforestation. It is traditionally used to treat inflammatory processes and liver and stomach disorders; and, as it is an aromatic species, it is used in personal hygiene. Phytochemical analysis of aerial and underground parts of *B. dracunculifolia* indicated the presence of essential oils, phenolic acids, flavonoids, diterpenes, triterpenes and glycosides. The essential oil extracted from aerial parts has great commercial value in the perfume industry. Sesquiterpenol (E)-nerolidol is considered a chemical marker of the oil with anti-ulcerative action. Through this review, it became clear that the presence of phenolic compounds such as phenolic acids: artepillin C, baccharin, vicenin, *p*-coumaric, dihydrocoumaric, ferulic, (E)-cinnamic, hydroxycinnamic, gallic acids, caffeic, and several caffeoylquinic derivatives; the flavonoids: naringenin, acacetin, dihydrokaempferol, isosakuranetin, kaempferide, pinobanksin, chrysin, apigenin, betuletol, galangin, pinocembrin, quercetin; of dracunculifosides type glycosides and pentacyclic triterpenoids: *Baccharis* oxide and friedelanol isolated from extracts and green propolis, guarantee this species intense anti-inflammatory, anti-ulcerogenic, antioxidant, antimicrobial activity, among others. However, research is needed to elucidate the mechanism of action and the relationship between compound structure and biological activity.
