# Supplementary material for: Ethnomedicinal, phytochemical and pharmacological investigations of Baccharis dracunculifolia DC. (ASTERACEAE)
Source: Front Pharmacol. 2022 Nov 28;13:1048688. doi: 10.3389/fphar.2022.1048688 (PMC9742423; doi:10.3389/fphar.2022.1048688)
Supplement: Supplementary file 1 [file DataSheet1.PDF]

Supplementary Material

Supplementary Figures

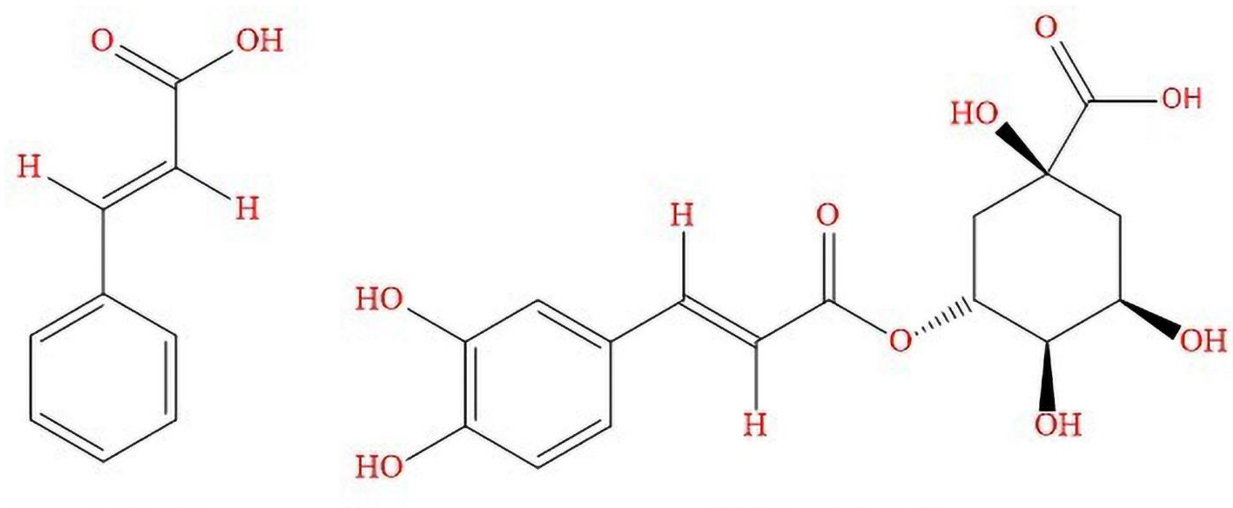

Cinnamic acid   Chlorogenic acid

Supplementary Figure 1. Derivatives of cinnamic acid and chlorogenic acid

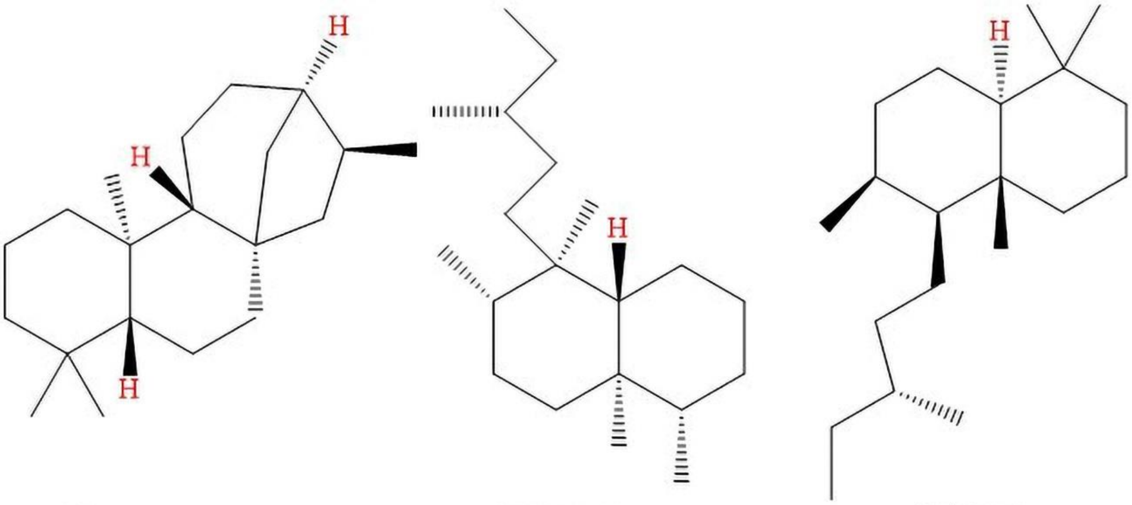

Kaurane   Clerodane   Labdane

Supplementary Figure 2. Diterpene class in the genus *Baccharis*

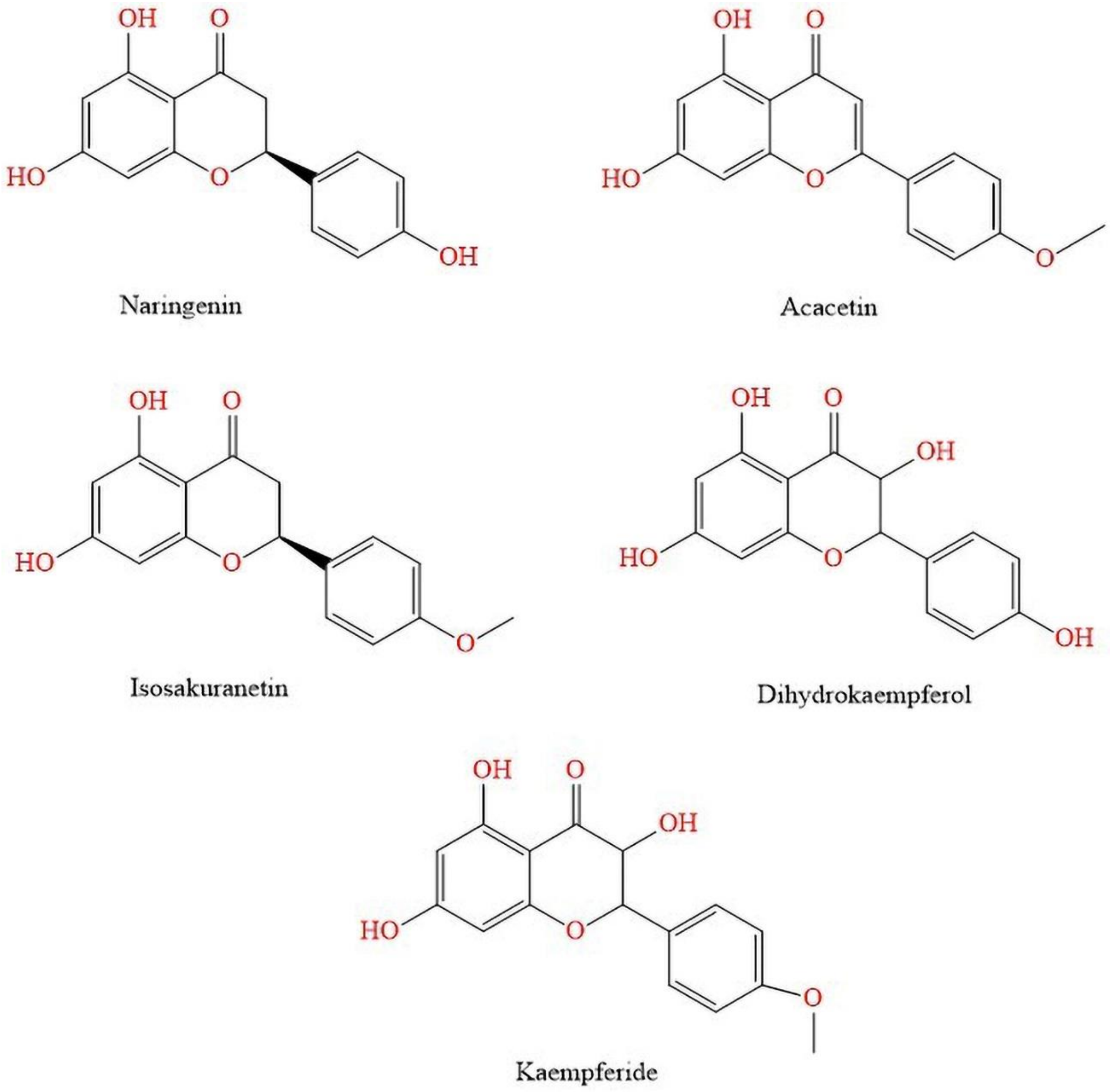

Supplementary Figure 3. Flavonoids found in *B. dracunculifolia* dried aerial parts in methanolic extract

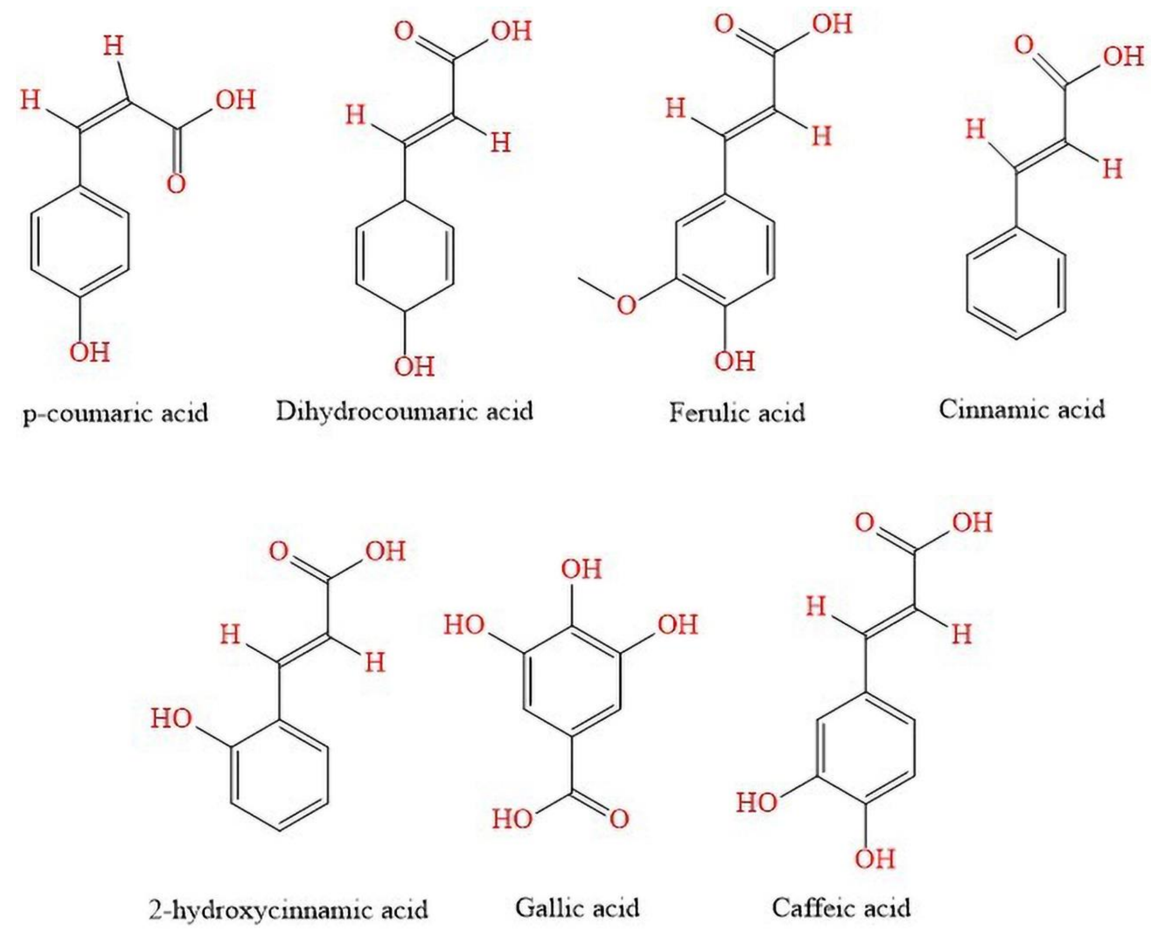

Supplementary Figure 4. Phenolic acids found in *B. dracunculifolia* hydroalcoholic extracts

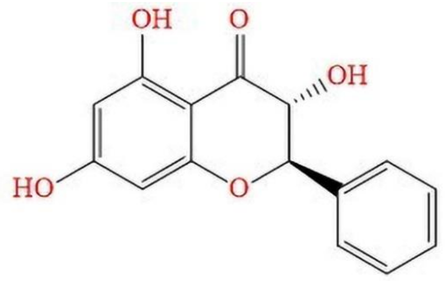

Pinobanksin

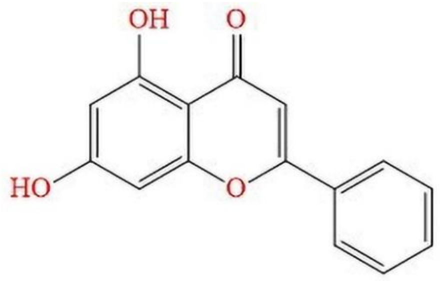

Chrysin

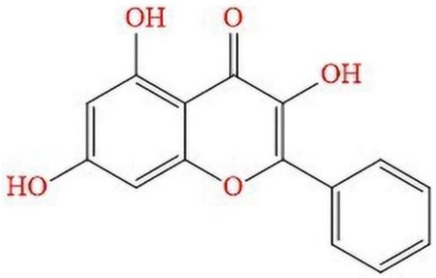

Galangin

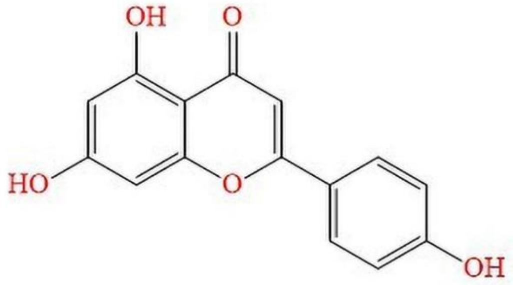

Apigenin

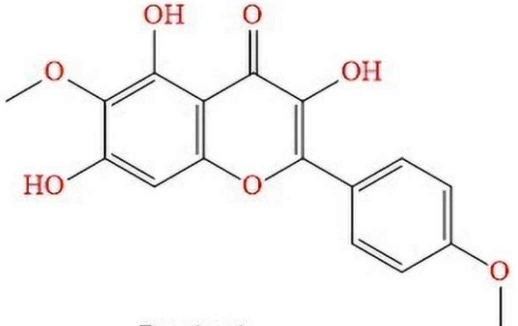

Betuletol

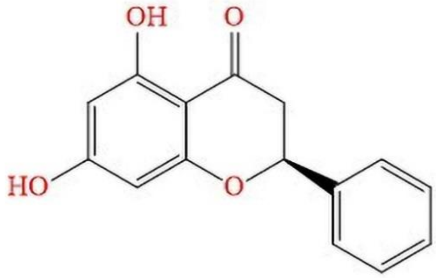

Pinocembrin

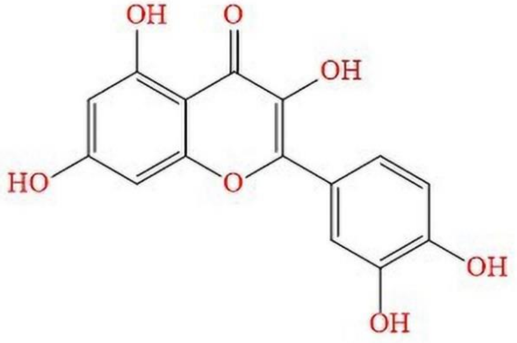

Quercetin

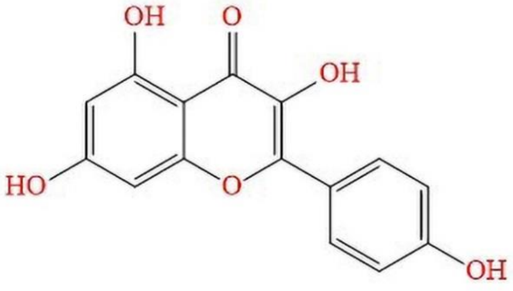

Kaempferol

Supplementary Figure 5. Flavonoids found in the hydroalcoholic extract of *B. dracunculifolia* leaves and green propolis.

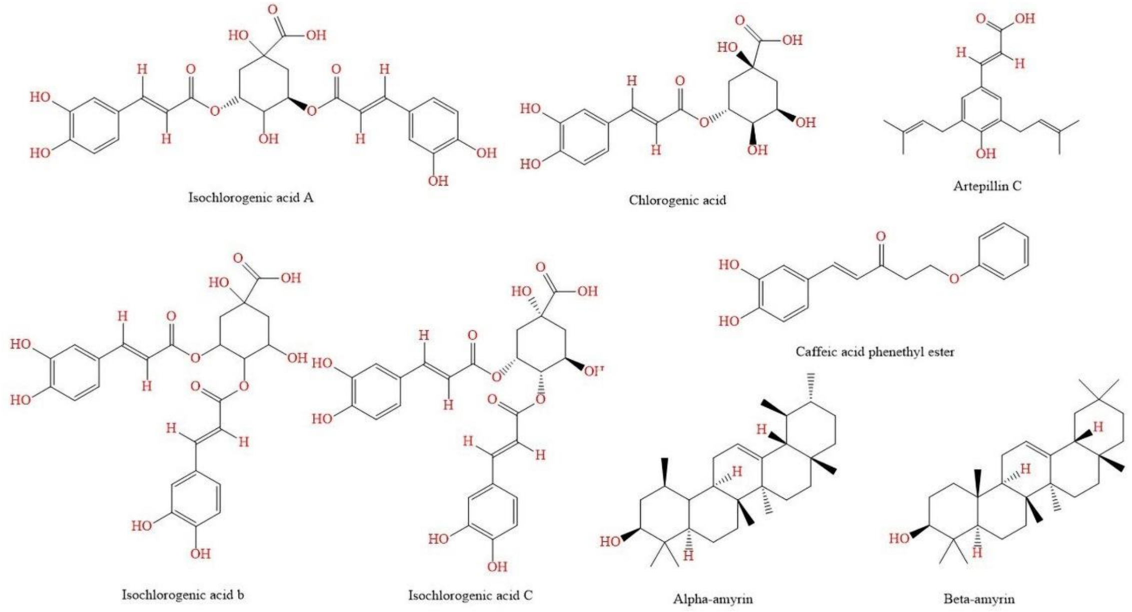

Supplementary Figure 6A. Chemical compounds found in Brazilian green propolis

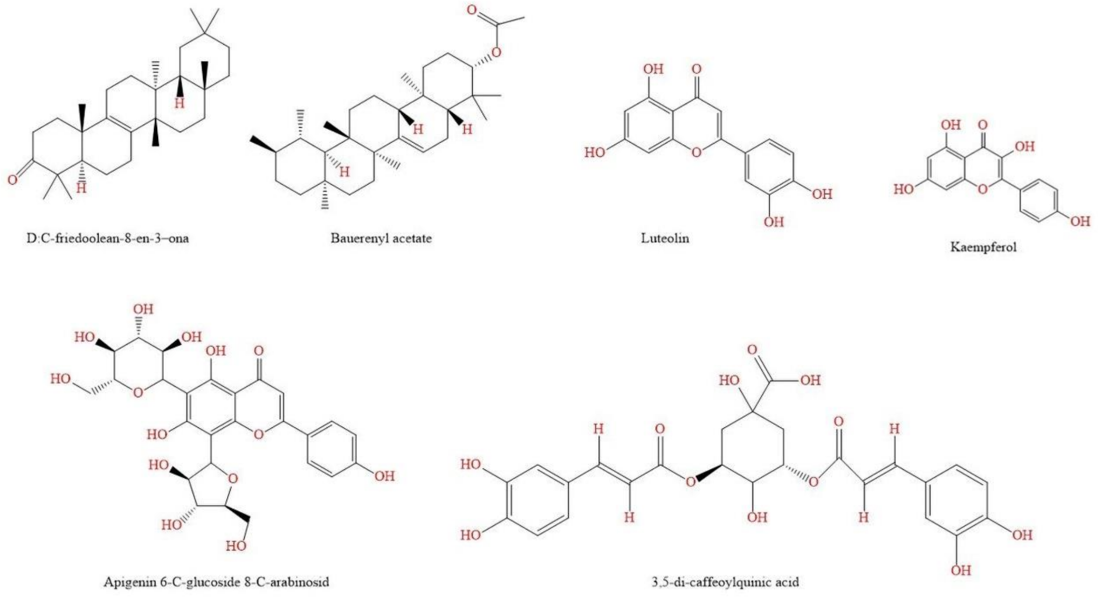

Supplementary Figure 6B. Chemical compounds found in Brazilian green propolis
